# Effects of Employee Sickness Presence on Customer Repurchase and Recommendation Intentions: The Role of Customer Affective Reactions

**DOI:** 10.1007/s10869-021-09764-1

**Published:** 2021-08-16

**Authors:** Carolin Dietz, Hannes Zacher

**Affiliations:** Institute of Psychology - Wilhelm Wundt, Leipzig University, Neumarkt 9-19 04109, Leipzig, Germany

**Keywords:** Sickness presence, Presenteeism, Customer service

## Abstract

Sickness presence can have important individual and organizational consequences, such as health deterioration or productivity loss. Additional risks, such as negative customer reactions, may be particularly relevant in the service sector. Based on affective events theory and appraisal theories, we hypothesize that employee sickness presence negatively impacts customer repurchase and recommendation intentions. Furthermore, we explore potential affective mechanisms of these effects, including disease avoidance, personal anger, moral outrage, post-consumption guilt, and customer compassion for the employee. We conducted four studies, including three experimental vignette methodology studies (*N*s = 227, 72, and 763) and a qualitative study (*N* = 54). In Study 1, employee sickness presence had negative effects on repurchase and recommendation intentions. Results of Study 2 show that customers experienced disgust, fear, anger, guilt, compassion, and indifference in response to sickness presence. In Study 3, anger explained the negative effects of employee sickness presence on repurchase and recommendation intentions, while appraisals of moral fairness were negatively related to both customer intentions. Finally, in Study 4, disgust and anger explained negative effects, while fear, guilt, and compassion explained positive effects of employee sickness presence on customer intentions. Appraisals of goal incongruence, reduced agency of the customer, and uncertainty were negatively related to customer intentions. The physical absence of the customer in the service encounter (phone call) mitigated the experience of disgust, fear, and anger, whereas it exacerbated feelings of compassion for the ill employee.

**Supplementary Information:**

The online version contains supplementary material available at 10.1007/s10869-021-09764-1.

Sickness presence, defined as working while being ill, has earned much interest due to its detrimental effects on employee health and performance (Lohaus & Habermann, 2019) and the related negative organizational consequences (Hemp, 2004; Schultz et al., 2009). However, research has largely neglected the context of sickness presence, such as specific occupations and industries. In particular, employee illness may not only affect the individual employee but also clients or customers (Ruhle et al., 2019). Indeed, a recent study showed that employees’ symptoms of a severe cold reduce customer intentions to recommend the service to others and to return to a hotel (i.e., repurchase a service; Correia Leal & Ferreira, 2019).

However, current understanding of the effects of employee sickness presence on customers is still limited, because we know only very little about the underlying processes of these effects. There is preliminary evidence for affective mechanisms, in that customers respond with feelings of anger and hostility to a sales person’s depersonalization (i.e., a dimension of the burnout syndrome which, for instance, involves a cynical attitude that may be expressed as unprofessional behavior toward clients). These affective mechanisms, in turn, have negative effects on customer attitudes (Nesher Shoshan & Sonnentag, 2019), which are related to customers’ behavioral intentions (Ajzen, 2018). Importantly, in the case of physical and possibly infectious diseases, there might be additional mechanisms, such as disease avoidance. This is relevant for practice because different forms of customer affective reactions require different organizational actions, such as apologies or compensation strategies (Antonetti, 2016).

In this article, we address the issue of affective reactions underlying the effects of employee sickness presence on customer repurchase and recommendation intentions by conducting three experimental vignette methodology studies (studies 1, 3, and 4) and one qualitative study (Study 2). In Study 1, we aim to replicate the negative effects of employee sickness presence on customers’ intentions to repurchase and recommend the service. We focus on an after-sales service that involves the installation of a product at the customer’s home. This is a prolonged service encounter and more intimate than an interaction at the check-in at a hotel, as the employee enters the customer’s home. Using a qualitative approach, in Study 2, we explore customers’ affective reactions to employee sickness presence in real-life service encounters. The affective reactions are then quantitatively examined as potential mechanisms of effects of employee sickness presence on customer repurchase and recommendation intentions in Study 3 (see Fig. 1). To this end, we use another experimental vignette methodology study with a scenario that describes the delivery of a parcel. This is a very brief, non-personal, and low affective service encounter and, therefore, represents the majority of daily life service interactions (Mattila & Enz, 2002). In Study 4, we additionally manipulated the physical presence/absence of the customer in the service encounter (holding duration and affective arousal constant) to examine the generalizability of the findings to other types of service encounters.

**Fig. 1:**
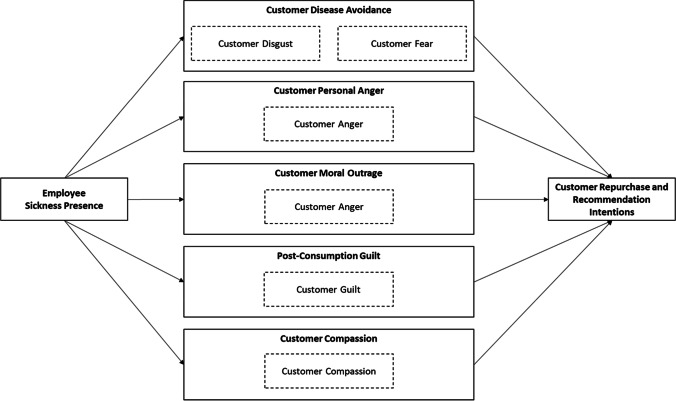
Proposed mechanisms underlying the effects of employee sickness presence on customer repurchase and recommendation intentions and their affective characteristics

Our research contributes to the emerging literature on the effects of sickness presence on customers by examining affective reactions to employee sickness presence in different service encounters. We also contribute to the conceptual development of this form of health-related behavior by combining affective events theory (Weiss & Cropanzano, 1996) with an appraisal theory perspective (Moors et al., 2013), the service literature, and the evolutionary and social psychological theoretical reasoning. Specifically, we conceive employee sickness presence as an affective event for customers, who subsequently show various cognitive reactions (i.e., appraisals) and affective reactions (i.e., emotions). These reactions may reflect evolutionary or social mechanisms, such as disease avoidance, and could influence customers’ intentions. Additionally, we aim to replicate the negative effects of an employee’s physical illness on customer repurchase and recommendation intentions in the hospitality sector (Correia Leal & Ferreira, 2019) and in the courier, express, and parcel industry, as this is a rapidly growing and economically important sector (Ducret & Delaître, 2013; Morganti et al., 2014). Finally, we contribute to a better understanding of variation in these effects depending on customers’ physical presence in the service encounter. Therefore, the results of our study may have important implications for practitioners and organizations in terms of health promotion, work design, absence policies, and customer relationship management.

In the following, we first examine the direct effects of employee sickness presence on customer repurchase and recommendation intentions (Study 1). Subsequently, we explore customers’ affective reactions in a service encounter with an ill employee (Study 2). Based on the results of the first two studies, we theorize on potential affective mechanisms that may explain the effects of employee sickness presence on customer repurchase and recommendation intentions. These mechanisms are examined and discussed in Study 3. Next, we propose a potential effect of the physical presence/absence of customers during the service encounter on the affective mechanisms and investigate this effect in Study 4. Finally, we summarize and discuss the results of all four studies.

## Sickness Presence as an Affective Event and Predictor of Customer Repurchase and Recommendation Intentions

A service encounter with an ill employee can be conceptualized as an (aversive) affective event for customers and, therefore, may reduce customer repurchase and recommendation intentions. Affective events theory (Weiss & Cropanzano, 1996) postulates that certain work events can lead to affective reactions of employees, which, in turn, influence attitudes toward the workplace. This assumption can also be applied to service encounters. Service encounters are events that can evoke affective reactions in customers, which subsequently may influence customer attitudes and evaluations of the service (McColl-Kennedy & Sparks, 2003). We assume that the interaction with an ill employee is an aversive affective event for the customers, as symptoms of illness are threat-signaling cues (Neuberg et al., 2011). Even though parasites are not always visible to the human eye, humans are highly vigilant for changes in physical appearance (e.g., skin lesions) and unusual, non-normative behavior (e.g., blowing one’s nose) caused by parasites (Kurzban & Leary, 2001; Neuberg et al., 2011). Detected symptoms of illness lead to a quick reaction with little consciousness and are characterized by feelings of disgust and fear, the activation of negative attitudes, and intentions for physical avoidance (Park et al., 2003; Pryor et al., 2004). Thus, employee sickness presence should have negative effects on customer repurchase and recommendation intentions. Indeed, employees’ symptoms of mental (i.e., burnout) and physical (i.e., common cold) illness have been shown to decrease favorable customer service evaluations such as service satisfaction, as well as recommendation and rebooking intentions (Correia Leal & Ferreira, 2019; Nesher Shoshan & Sonnentag, 2019; Söderlund, 2017). However, the negative effect of burnout symptoms on customer attitudes was explained by customer feelings of anger and hostility (Nesher Shoshan & Sonnentag, 2019). Thus, there might be additional affective responses to employee sickness presence besides customer disgust and fear of contagion.

The link between events and affective reactions reflects processes of affect instigation, which can be described and differentiated by appraisal theories of emotions (Weiss & Beal, 2005). Appraisal theorists have begun to investigate the role of appraisals for the development of emotions a long time ago (Frijda, 1986; Lazarus, 1991). From an appraisal theory perspective, the appraisal of an event determines the intensity and quality of feelings, action tendencies, and behavior (Lazarus, 1991; Moors et al., 2013). At least three appraisal criteria are relevant for the differentiation of affective reactions (e.g., Frijda, 1986; Lazarus, 1991). Goal congruence is the evaluation of the fairness of an outcome in terms of social norms or personal standards and determines the valence and the intensity of the affective reaction (Ma et al., 2013). Agency differentiates between affective reactions focusing on the self, another person, or a third entity (Moors et al., 2013). Certainty determines whether the outcome is known or certain and, therefore, differentiates affective reactions related to the outcome from anticipatory reactions (Ma et al., 2013). Thus, customers may appraise employee sickness presence as unfair, undeserved, or incongruent to one of their goals (e.g., obtaining information, staying healthy, corporate responsibility), which can elicit feelings with a negative valence. However, appraisals of responsibility for and certainty of the goal incongruence may differ between customers and, in consequence, result in different negative feelings (Ma et al., 2013), service attitudes, and specific behavioral intentions (Ajzen, 2018). Disgusted customers may blame objects (e.g., germs) or circumstances (e.g., flu season), whereas angry customers should hold others, such as the employee (e.g., unprofessional, irresponsible behavior) or the management (e.g., corporate irresponsibility), responsible for the goal incongruence. In contrast, customers who predominantly consider themselves responsible (e.g., service interaction was self-induced) should feel guilty, whereas fear can be related to various appraisals of agency, but differs from the other affective reactions in terms of high uncertainty about the outcome of the situation (e.g., potentially unhelpful service, unknown risk of contagion).

In summary, we postulate that a service encounter with an ill employee is an aversive affective event that negatively influences customer repurchase and recommendation intentions. The elicited affective reactions are determined by customers’ appraisals of the service encounter, are characterized by different emotions, and can evoke different action tendencies. Therefore, it is important to explore customers’ affective reactions to employee sickness in greater depth.

- *Hypothesis 1*: Service employee sickness presence has a negative effect on customer (a) repurchase and (b) recommendation intentions.

- *Research Question 1*: Which affective reactions do customers have within a service encounter with an ill employee?

## Study 1

### Method

To test our hypothesis, we conducted an experimental vignette methodology study. Experimental vignette methodology studies entail the presentation of realistic scenarios to participants, in which the independent variable(s) are manipulated, and the dependent variables, such as intentions or attitudes, are assessed (Aguinis & Bradley, 2014). The vignettes we used (Table 1) were validated in an independent pilot study (*N* = 10 participants recruited through personal contacts). Overall, the results of the pilot study suggest that the two scenarios were sufficiently clear and distinct. Thus, we used the same scenarios in the main study. Detailed information is provided in Table S1 in the Supplementary information at https://osf.io/368b7/.

**Table 1:** Scenarios of Studies 1, 3, and 4

Study 1	Scenario 1a/1b You purchased a technical device/product. The service of setting up/installing and commissioning the device are included in the scope of delivery. The scheduled date of delivery is complied with and the product works as expected. During setup/installation, you chat with the employee of the supplier and learn that he volunteered to carry out your delivery **even though he is in poor health**
Study 3	Scenario 2a/2b You are expecting a parcel. On the scheduled delivery date, the Star Express courier rings your doorbell. He greets you with **a hoarse and barely audible voice as well as a pale face**. He hands you the parcel and asks you to sign the confirmation of receipt. Then, he says goodbye
Study 4	Scenario 3a/3b and 4a/4b You urgently want to open an account at the MARO Bank. On the webpage, you have already been able to quickly and easily find out about the fair offers of the MARO Bank. However, you still have a few questions about the conditions of the account model you have chosen, for which you have not found any information on the webpage. *For this reason, you go to a nearby branch of the MARO Bank./For this reason, you call MARO Bank’s toll-free hotline* *Directly, a bank employee asks you to approach a consulting desk./Directly, you are put through to a bank employee.* He greets you politely and asks you **with a hoarse, scratchy voice** about your request. You want to know how much a credit card costs per year and whether you can use it to withdraw money abroad for free. The bank employee kindly explains to you that a credit card costs 12 euros per year and that you can of course use it to withdraw money abroad free of charge. **While the bank employee is talking, he has to cough very hard again and again.** You say that you would like to think again about the credit card and *the bank employee kindly offers you an information brochure with the most important details./the bank employee kindly describes where you can find an info brochure with the most important details on the homepage.* **Afterwards, he apologizes and blows his nose** You thank him and ask him at what conditions you could have a bank overdraft. The bank employee says with a husky voice that the MARO Bank is guided by the current interest rate level when setting the bank overdraft rates. So the interest rate for up to 5000 euros overdraft is currently 0.00%. Also this time, the bank employee obligingly *offers you an information brochure. / explains to you where you can find an information brochure.* **When talking, he has to cough heavily again and again and also pulls up his nose several times.** *You thank him for the helpful conversation/You thank him for the helpful phone call* and look at your watch, as you still have an appointment. You see that the conversation lasted about 10 min. *Then you say goodbye and leave./Then you say goodbye and hang up*

To manipulate employee sickness presence, Scenarios 1b, 2b, 3b, and 4b additionally include cues about employee’s health impairments (in bold). Scenarios 3 and 4 differ with regard to physical presence/absence (in italics)

#### Participants and Procedure

Participants were recruited via announcements in a German university, recruited through requests via social networks and recruiting platforms, as well as through personal contacts. The online study was completed by 309 participants. We excluded one participant who was below 18 years and 81 participants because they failed an attention check (i.e., after the outcome measures were collected, the participants were asked whether the employee described in the scenario was ill; please see sensitivity analyses below). The final sample consisted of *N* = 227 participants, including 151 women (66.5%) and 76 men (33.5%). The average age of participants was 29.25 years (*SD* = 8.71) and ranged from 18 to 60 years. Participants worked in various industries, such as medicine and civil service, education, and engineering (detailed information is provided in Table S2). A small share of the sample indicated to have no profession (18.1%; e.g., university students).

First, participants were asked to carefully read and imagine one of the two scenarios (Table 1). The scenario was randomly selected and assigned to participants. Afterwards, participants were asked to indicate their intentions to repurchase and recommend the described service and to provide demographic information. Scenario 1a (no sickness presence) was rated by 126 participants, and 101 participants rated Scenario 1b (sickness presence). The two groups were demographically very similar (Table S2).

#### Measures

To measure customer repurchase and recommendation intentions, we used two adapted and translated items from the Client Satisfaction Questionnaire (CSQ-8; Attkisson & Greenfield, 1994). *Repurchase intention* was measured with the item, “Would you come back to this service provider for this kind of service?” and *recommendation intention* was measured with the item, “Would you recommend this service to a friend?” Participants responded on 5-point scales ranging from 1 (*very unlikely*) to 5 (*very likely*). The correlation between these two items was *r* = 0.90 (*p* < 0.001) and, overall, the pattern of results was not substantially different to the results reported below when we used an average score of the two items in additional analyses.

#### Statistical Analyses

We tested our hypothesis with a one-way between-subjects analysis of variance (ANOVA) using a 95% significance level.

### Results and Discussion

Testing the assumptions of the ANOVA revealed violations of normality and homogeneity of variances (Table S3). Thus, we conducted a more robust Welch’s ANOVA (Delacre et al., 2019). In line with Hypotheses 1a and 1b, there were significant main effects of employee sickness presence on customer repurchase intention, Welch’s *F* (1, 183.84) = 22.34, *p* < 0.001, *η*^*2*^ = 0.10, and recommendation intention, Welch’s *F* (1, 189.44) = 15.35, *p* < 0.001, *η*^*2*^ = 0.07. Repurchase and recommendation intentions were rated higher in Scenario 1a without a cue of employee sickness presence (*M* = 4.18, *SD* = 0.92 for repurchase intention; *M* = 4.08, *SD* = 0.96 for recommendation intention) than in Scenario 1b, which included such a cue (*M* = 3.50, *SD* = 1.21 for repurchase; *M* = 3.50, *SD* = 1.20 for recommendation intention).

Additionally, we conducted a sensitivity analysis using a Mann–Whitney-*U*-test, which is a non-parametric method. The distributions of customer repurchase intention (Kolmogorov–Smirnov-*Z* = 1.94, *p* = 0.001) and recommendation intention (Kolmogorov–Smirnov-*Z* = 1.63, *p* = 0.010) differed between both scenarios. Thus, we interpreted the sum of the rank, but not the median. There were significant differences in repurchase intention between Scenario 1a (*M*_Rank_ = 16,466.50) and Scenario 1b (*M*_Rank_ = 9411.50), *U* = 4260.50, *Z* =  − 4.50, *p* < 0.001, and also in recommendation intention between Scenario 1a (*M*_Rank_ = 16,104.00) and Scenario 1b (*M*_Rank_ = 9774.00), *U* = 4623.00, *Z* =  − 3.72, *p* < 0.001.

Sensitivity analyses with a sample that also included the 81 participants who failed the attention check revealed comparable results. There were significant main effects of employee sickness presence on customer repurchase intention, Welch’s *F* (1, 297.91) = 22.75, *p* < 0.001, *η*^*2*^ = 0.07, and recommendation intention, Welch’s *F* (1, 300.55) = 15.32, *p* < 0.001, *η*^*2*^ = 0.05. Repurchase and recommendation intentions were rated higher in Scenario 1a without a cue of employee sickness presence (*M* = 4.11, *SD* = 0.96 for repurchase; *M* = 3.99, *SD* = 1.01 for recommendation) than in Scenario 1b, which included such a cue (*M* = 3.52, *SD* = 1.18 for repurchase; *M* = 3.49, *SD* = 1.21 for recommendation).

Overall, these results support our assumption that employee sickness presence has negative effects on customer repurchase and recommendation intentions (Hypothesis 1a and 1b; for an overview of the results, see Table 2). They replicate findings of negative effects of employee sickness presence on customer return and recommendation intentions in public service encounters (Correia Leal & Ferreira, 2019) and suggest that these effects might be transferable to other types of service encounters. However, the underlying processes are still not clear. Thus, we addressed our Research Question 1 in the next study.

**Table 2:** Summary of direct and indirect effects of the employee sickness presence on customers’ affective reactions and repurchase and recommendation intentions

	Study 1	Study 3	Study 4	
	Unconditional effect	Unconditional effect	Unconditional effect	Interaction with Physical Presence/Absence^a^
Direct Effects of SP on				
Repurchase Intention	Negative	Negative	Negative	n.s
Recommendation Intention	Negative	Negative	Negative	Weakening
Disgust	/	Positive	Positive	Weakening
Fear	/	Positive	Positive	Weakening
Anger	/	Positive	Positive	Weakening
Guilt	/	Positive	Positive	n.s
Compassion	/	Positive	Positive	Strengthening
Indirect effects of SP on Rep through				
Disgust	/	n.s	Negative	Weakening
Fear	/	n.s	n.s	n.s
Anger	/	Negative	Negative	Weakening
Guilt	/	n.s	Positive	n.s
Compassion	/	n.s	Positive	Strengthening
Indirect effects of SP on Rec through				
Disgust	/	n.s	Negative	Weakening
Fear	/	n.s	Positive	Weakening
Anger	/	Negative	Negative	Weakening
Guilt	/	n.s	n.s	n.s
Compassion	/	n.s	Positive	Strengthening

^a^Physical Presence/Absence of the customer during the service encounter: presence (0), absence (1); *SP*, Sickness Presence: not present (0), present (1); *Rep*, Repurchase Intentions; *Rec*, Recommendation Intentions; *n.s.*, effect not significant at *α* .05

## Study 2

### Method

#### Participants and Procedure

We used a qualitative study to explore Research Question 1. Participants were recruited via announcements in a German university, recruited through requests via social networks and recruiting platforms, and through personal contacts. In total, 155 participants responded to our online questionnaire, in which we used an adapted form of the day reconstruction method (Kahneman et al., 2004). Of these, 69 participants remembered a recent service encounter with an ill employee from their real life. We excluded 64 participants, who did not remember such a situation, and 22 participants, who did not respond at all to the question. Participants were instructed to remember one of their recent real-life service encounters as accurately as possible, in which the service employee seemed to be ill. To facilitate this process, we initially asked about the context of this encounter (e.g., when the event was, where it took place). Afterwards, an open-ended question was presented, asking participants how they felt about the service employee being ill.

After screening the responses to the open-ended question, we excluded 11 participants, who did not answer the actual question and four participants because of very brief, nonsensical responses. The final sample thus comprised of *N* = 54 participants (34.8%). The sample consisted of 41 women (75.9%), 12 men (22.2%), and one person indicating their gender as other (1.9%). The average age of participants was 28.43 years (*SD* = 10.98) and ranged from 18 to 66 years. About one-third of participants (35.2%) indicated to be employed. They worked in a variety of professions such as medicine, engineering, educational professions, academia, and economics. The other participants were mainly trainees or students (53.7%). A minority of participants was unemployed (5.6%), self-employed (3.7%), or retired (1.9%).

#### Qualitative Analyses

We used the software MAXQDA (VERBI Software, 2019) to conduct a thematic analysis (Braun & Clarke, 2006), which is a qualitative method to identify, analyze, and report repeated patterns of meaning (themes) in qualitative data. In a first step, the first author familiarized herself with the data, which involved the reading and re-reading of the responses to the open-ended question about participants’ (affective) reactions as well as noting initial ideas. Second, the first author used the responses to systematically generate initial codes and collated relevant data to each code. Third, the first author collated codes into potential themes and, fourth, checked if the themes fit with the coded extracts and generated a thematic map of the analysis. The first author also examined the data for differences and commonalities within and across themes. In the next step, the first author refined the themes, generated names and definitions, and selected exemplary codes for each theme (Table 3). Based on this coding scheme, two raters, who were student assistants and familiar with the research question, independently coded participants’ responses to the open-ended question about the illness of the employee. The raters were free to assign multiple codes to each response. All original and translated responses as well as the ratings of the two raters are presented in Table S4.

**Table 3:** Coding scheme and examples for respondents’ affective reactions to employees’ illness (Study 2)

	C	Themes	Definition	Exemplary codes	Examples
Negative affective reactions	1	Disgust	Disgust is typically experienced as revulsion, can be accompanied by nausea, and often goes along with intentions to physically avoid the eliciting stimulus (Oaten et al., 2009)	Revolting, repulsive	“Disgusting. Not professional.”
	2	Fear	Fear is an aversive affective state in response to an identifiable threat and goes along with an urge to defend oneself, primarily by distancing from the eliciting stimulus (Öhmann, 2008)	Threatening, deterrent, being afraid	“I was unsettled, because I visited a new hair stylist and was afraid that the illness of the stylist would influence the haircut.” “[…] And of course I was a bit afraid that she might infect me and not wash her hands enough or something …”
	3	Anger	Anger, which is the affective reaction to the appraisal of responsibility for wrongdoing or unfair treatment by others (Gibson and Callister 2010)	Outrage, irritation, impertinence, impudence	“I did not feel that she was fully capable and I did not get the service I hoped for.” “[…] Outrage about the company.”
	4	Guilt	Guilt, an unpleasant feeling that arises after one’s personal action may have transgressed an internal moral, societal, or ethical standard (Baumeister et al., 1994; Kugler and Jones 1992)	Feeling bad, ashamed, guilty	“[I] felt guilty for taking up his time. […]” “Somewhat unpleasant, because I was sitting at home healthy and the service provider was hastily trying to deliver parcels.”
	5	Other negative affective reactions	Other affective reactions with a negative valence		“[I feel] sad.” “[I feel] bad.”
Ambiguous affective reactions	6	Compassion	Compassion arises when people recognize another person’s suffering, which can subsequently motivate intentions to help (Goetz et al., 2010)	Compassion, pity, being sorry, regrettable	“I was sorry and I did not know exactly what would have been appropriate to do or say.” “I felt pity, […], especially because in our society people are not resting enough and keep going even though the body tells one that it is not working anymore.”
Positive affective reactions	7	Positive affective reactions	Affective reactions with a positive valence		“[…] I was glad to have been served nicely.” “I was astonished that someone would do this job, even though it obviously takes a lot out of him. […] So I was rather impressed by how quickly everything was done […]”
Neutral reactions	8	Indifference	Conscious perception of employee’s illness; objective and neutral reaction; non-affective or very low intensive affective reactions	Not as bothering, indifferent	“I did not experience this as too bad. Wages are paid by the hour and I don’t need to stay home because of a runny nose.” “… not as bothering – everyone has to deal with a cold once in a while.” “Not bad, as it was something mental and the person already recovering.”
Miscellaneous reactions	9	Miscellaneous reactions	Reactions that do not fit in the scheme		“[The employee] coughed” “I told him to take a day of sick leave instead. The bosses are very obliging.”

*C* coding

### Results

Participants’ written responses to the open-ended question about their feelings had an average length of 67.91 characters (*SD* = 73.54), ranging from 7 to 355 characters. Eight themes of (affective) reactions to employees being ill became evident from the thematic analysis. These were (1) disgust, (2) fear, (3) anger, (4) guilt, (5) other negative affective reactions, (6) compassion, (7) positive affective reactions, and (8) indifference. Besides the eight main themes, there were reactions that did not fit into the coding scheme and were categorized as (9) miscellaneous. While the raters assigned some responses of the participants to only one theme, other responses were assigned to multiple themes. Rater 1 identified 63 (affective) reactions in the 54 responses of the participants, and Rater 2 identified 64 (affective) reactions (Table 4). The intercoder agreement between the two raters was good, *κ* = 0.78 (95% CI, 0.67 to 0.90), *p* < 0.001. Exemplary responses for each theme are presented in Table 3.

**Table 4:** Frequencies of affective reactions and respondents’ thoughts about the ill employee separated by affective reaction (Study 2)

	Rater 1	Rater 2		Rater 1	Rater 2
	*n* (%)	*n* (%)	Thoughts per affective reaction	*n* (%)	*n* (%)
Disgust	1 (1.6)	2 (3.1)	Unspecific causes	/	1 (50.0)
			Miscellaneous	1 (100.0)	1 (50.0)
Fear	6 (9.5)	5 (7.8)	Causes in social/economic system	1 (20.0)	/
			Unspecific causes	1 (20.0)	1 (25.0)
			Consequences for customers	3 (60.0)	3 (75.0)
Anger	4 (6.3)	5 (7.8)	Causes in social/economic system	2 (50.0)	2 (40.0)
			Unspecific causes	1 (25.0)	1 (20.0)
			Consequences for customers	1 (25.0)	1 (20.0)
			Consequences for employee	/	1 (20.0)
Guilt	2 (3.2)	5 (7.8)	Causes in social/economic system	/	1 (14.3)
			Causes in work characteristics	2 (50.0)	2 (28.5)
			Individual causes of the employee	1 (25.0)	3 (42.9)
			Unspecific causes	1 (25.0)	/
			Problem-oriented coping	/	1 (14.3)
Other negative affective reactions	12 (19.0)	11 (17.2)	Causes in social/economic system	1 (5.0)	3 (20.0)
			Causes in work characteristics	2 (10.0)	1 (6.67)
			Unspecific causes	2 (10.0)	3 (20.0)
			Consequences for customers	3 (15.0)	3 (20.0)
			Consequences for employee	4 (20.0)	/
			Problem-oriented coping	2 (10.0)	/
			Miscellaneous	6 (30.0)	5 (33.33)
Compassion	20 (31.7)	19 (29.7)	Causes in social/economic system	2 (10.0)	2 (9.1)
			Causes in work characteristics	5 (25.0)	5 (22.7)
			Individual causes of the employee	2 (10.0)	3 (13.6)
			Unspecific causes	/	1 (4.5)
			Consequences for employee	3 (15.0)	2 (9.1)
			Problem-oriented coping	4 (20.0)	4 (18.2)
			Miscellaneous	4 (20.0)	5 (22.7)
Positive affective reactions	1 (1.6)	3 (4.7)	Causes in work characteristics	1 (50.0)	1 (33.3)
			Individual causes of the employee	1 (50.0)	/
			Problem-oriented coping	/	1 (33.3)
			Miscellaneous	/	1 (33.3)
Indifference	7 (15.9)	10 (15.6)	Causes in social/economic system	1 (7.7)	/
			Causes in work characteristics	2 (15.4)	1 (9.1)
			Individual causes of the employee	1 (7.7)	/
			Unspecific causes	/	1 (9.1)
			Consequences for customers	/	1 (9.1)
			Consequences for employee	1 (7.7)	/
			Problem-oriented coping	1 (7.7)	1 (9.1)
			Miscellaneous	7 (53.8)	7 (63.6)
Miscellaneous	7 (11.1)	4 (6.3)	Unspecific causes	1 (16.7)	1 (50.0)
			Consequences for customers	2 (33.3)	1 (50.0)
			Problem-oriented coping	2 (33.3)	/
			Miscellaneous	1 (16.7)	/

*N* = 54 respondents answered the open-ended question about their affective reactions regarding employee’s illness. Rater 1 identified 63 (affective) reactions within these responses, while ﻿Rater 2 identified 64 (affective) reactions. *N* = 50 respondents answered the open-ended question about their thoughts regarding the ill employee. Rater 1 identified 63 thoughts within these responses, while ﻿Rater 2 identified 61 thoughts

#### Negative Affective Reactions

There was a variety of affective reactions in the responses that had a negative valence. The first author categorized these reactions into five themes and labelled them disgust, fear, anger, guilt, and other negative affective reactions. *Disgust* is typically experienced as revulsion, can be accompanied by nausea, and often goes along with intentions to physically avoid the eliciting stimulus (Oaten et al., 2009). *Fear* is an aversive affective state in response to an identifiable threat and goes along with an urge to defend oneself, primarily by distancing from the eliciting stimulus (Öhmann, 2008). In this study, the first author defined revolting feelings and repulsion as exemplary codes for the theme disgust, while the exemplary codes for the theme fear were feelings of threat, deterrence, and being afraid. *Anger* is the affective reaction to the appraisal of responsibility for wrongdoing or unfair treatment by others (Gibson & Callister, 2010). Exemplary codes for this theme are outrage, irritation, impertinence, and impudence. *Guilt* is an unpleasant feeling that arises after one’s personal action may have transgressed an internal moral, societal, or ethical standard (Baumeister et al., 1994; Kugler & Jones, 1992). Exemplary codes for this theme are feeling bad and ashamed about one’s own actions. Furthermore, there were other negative affective reactions that did not fit into the themes of disgust, fear, anger, and guilt.

#### Ambiguous Affective Reactions

There were affective reactions that were categorized into one theme that was named compassion by the first author. *Compassion* arises when people recognize another person’s suffering, which can subsequently motivate intentions to help (Goetz et al., 2010). Whether this reaction is perceived as pleasurable or not is equivocal (Condon & Feldman Barrett, 2013) and, therefore, the valence of these affective reaction is ambiguous. In this study, exemplary codes for the theme compassion are feelings of sympathy, pity, and empathetic concern.

#### Positive Affective Reactions

There were also affective reactions to employee’s illness with a positive valence. As these reactions were diverse single entries, the first author collated them to one theme that was named positive affective reactions. Rater 1 assigned the theme positive affective reactions to 1 (1.6%) response, and Rater 2 assigned it to 3 (4.7%) responses (Table 4).

#### Neutral Reactions

There were also some neutral, non-affective, or low affective reactions. The first author categorized these reactions into one theme that was named indifference. *Indifference* was defined as an objective or neutral reaction to the conscious perception of employee’s illness, which is not affective or has very low affectivity. Exemplary codes for the theme indifference were indifference and not bothering.

#### Miscellaneous Reactions

There were reactions that did not fit into the coding scheme. These reactions were collated to the theme miscellaneous.

#### Additional Analysis

The reported affective reactions were partly accompanied by thoughts about the service encounter. Such cognitions (i.e., appraisals) could offer a better understanding of customers’ affective reactions (Moors et al., 2013). Thus, we conducted another thematic analysis similar to the one described above with data of an open-ended question that was presented after the question about participants’ feelings. Participants were asked to indicate their thoughts about the ill employee. Again, the first author developed a coding scheme (Table S5). Based on this scheme, two student assistants independently coded the responses of the participants (Table S4). All original and translated responses can be seen in Table S4. 

Participants’ written responses to the open-ended question about their thoughts had an average length of 77.56 characters (*SD* = 49.91), ranging from 8 to 234 characters. Seven themes of thoughts about the ill employee became evident from the thematic analysis. These were (1) causes for sickness presence in the social/economic system, (2) causes for sickness presence in the work characteristics of the employee, (3) the employee’s individual causes for sickness presence, (4) unspecific causes for sickness presence, (5) consequences of sickness presence for the customers, (6) consequences of sickness presence for the employee, and (7) problem-oriented coping. Beside the seven themes, there were reactions that did not fit into the coding scheme and were categorized as (8) miscellaneous. Rater 1 identified 63 appraisals in the 50 responses of the participants, and Rater 2 identified 61 appraisals (Table S4). The intercoder agreement between the two raters was good, *κ* = 0.83 (95% CI, 0.72 to 0.94), *p* < 0.001. Exemplary responses for each theme are presented in Table S5. In general, participants with the same affective reaction reported a variety of appraisals rather than a systematic pattern (Table 4).

### Discussion

Research Question 1 addressed customers’ affective reactions in a service encounter with an ill employee. Although some customers showed indifference, others had a variety of negative, ambiguous, or positive affective reactions to employee sickness presence. Thus, a service encounter with an ill employee seems to be an affective event for the customer. The identified negative affective reactions of disgust, fear, and anger could explain the negative effects of employee sickness presence on customer repurchase and recommendation intentions, as they go along with undesired action tendencies to avoid or harm the responsible entity (e.g., service provider; Funches, 2011; Oaten et al., 2009). Indeed, previous research has found anger to mediate the negative effects of employee sickness presence on customer attitudes (Nesher Shoshan & Sonnentag, 2019). However, the role of guilt and compassion is equivocal in terms of answering our research question. Guilt can inhibit or foster intentions for reparative actions like repurchase (Dahl et al., 2005; Ki et al., 2017). Compassion fosters prosocial behaviors (Pfattheicher et al., 2019) and prosocial lying (Lupoli et al., 2017), but also third-party punishment (Pfattheicher et al., 2019). Thus, it remains unclear how customers feeling guilty or having compassion for the employees affect repurchase and recommendation intentions. Additionally, positive affective reactions were very rare, and it is not clear how these reactions may explain the negative effects of sickness presence on customers’ intentions, as positive affective reactions should go along with beneficial action tendencies (Keiningham et al. 2018).

In Study 2, we also found that customers had different cognitive appraisals about the ill service employee. Customers’ thoughts about causes for sickness presence may reflect questions about the responsibility (i.e., agency) for the circumstances of the service encounter. Considerations about the consequences of sickness presence could be evaluations of the event in terms of fairness and goal congruence. One major topic was the risk of contagion. This supports our assumption that customers can recognize employees’ sickness and include this particular condition in their appraisals about the service encounter. Furthermore, thoughts about problem-oriented coping can be seen as action tendencies. However, the different affective reactions were not associated with a clear pattern of appraisals as suggested by appraisal theories (Moors et al., 2013). Thus, customers might have only a limited or unsystematic access to their appraisals in response to employee sickness. Indeed, appraisal theorists have stated that appraisals can involve complex, conscious processing but also simpler non-conscious processing, including primitive processing of sensory properties of stimuli and automatic priming or schematic cognitions (Roseman & Smith, 2001).

In the following, we integrate the appraisal theory perspective (Moors et al., 2013) with evolutionary and social psychological reasoning about reactions to disease to further elaborate on the origins and consequences of the identified discrete negative and ambiguous affective reactions and their accompanying thoughts. Specifically, we next discuss five theoretical mechanisms that might explain the effects of employee sickness presence on customers’ intentions (for a summary, see Fig. 1). These are disease avoidance, personal anger, moral outrage, post-consumption guilt, and customer compassion for the employee.

#### Sickness Presence as a Trigger for Disease Avoidance

Employee symptoms of illness could trigger a disease avoidance mechanism in customers, which might be characterized by feelings of disgust and fear as well as appraisals of a health threat (goal incongruence) caused by symptoms of illness (agency of objects or circumstances; Lazarus, 1991). According to the literature on disease avoidance, humans have developed a behavioral immune system that complements the immune system of the body to avoid contagion rather than just fight pathogens after infection (Neuberg et al., 2011). This disease avoidance mechanism includes cognitive (negative attitudes) and affective (disgust, fear) reactions to trigger the adaptive behavior of physical avoidance and anti-social reactions (Park et al., 2003). Thus, when employee illness evokes this evolutionary mechanism, customers should be disgusted or afraid and want to avoid the employee (i.e., not repurchasing or recommending the service provider to close others).

#### Sickness Presence as a Trigger for Personal Anger

Employee sickness presence may also trigger personal anger in customers, which is the mechanism arising when people recognize the thwarting of their own interests and at being personally treated unfairly (goal incongruence; Batson et al., 2007). According to fairness theory (Folger & Cropanzano, 1998), perceptions of unfairness are formed in a largely unconscious, automatic process. Research on service failures shows that a poor service, employee mistakes, or unprofessional behavior can cause costumer anger (Funches, 2011). Personal anger, in turn, can motivate actions to restore fairness, such as punishing the harm-doer (agency of others; Batson et al., 2009) and can also negatively impact customer service attitudes and behavioral intentions (Antonetti, 2016).

#### Sickness Presence as a Trigger for Moral Outrage

Employee sickness presence could further elicit customer moral outrage, that is, a mechanism characterized by the feeling of anger and provoked by the appraisal that a moral standard (e.g., fairness) has been violated (goal incongruence; Batson et al., 2007). Even though personal anger and moral outrage are experienced as anger, the appraisals of these two mechanisms are different (Batson et al., 2009; Hechler & Kessler, 2018). Customers may appraise the service provision by an ill employee as a service failure and unfair treatment of themselves. However, the assessment of fairness also involves ethical and moral standards (Folger & Cropanzano, 1998), which can be relevant for an individual just as threats to personal well-being (Gibson & Callister, 2010). Thus, customers may see the employees as targets of unfairness. Previous research has shown that costumers can react with feelings of anger to perceived unfairness of corporate behavior such as irresponsibility toward workers (i.e., abuse of child labor; Grappi et al., 2013) and, consequently, have intentions to bad mouth against the company (Antonetti & Maklan, 2016).

#### Sickness Presence as a Trigger for Post-consumption Guilt

Employee sickness could evoke customer post-consumption guilt, which involves feeling guilty after a consumption decision (Antonetti & Maklan, 2014). In contrast to personal anger and moral outrage, customers may attribute the responsibility for the aversive service encounter (goal incongruence) internally (agency of the customer). Research shows that customers react with guilt to varying transgressions in consumption such as purchase of unethical products (Antonetti & Maklan, 2014), self-caused service failure (Soscia, 2007), or being rude to a salesperson (Dahl et al., 2003). The organizational consequences of costumer guilt are equivocal. Guilt is associated with confession, apology, and attempts of reparative action (Baumeister et al., 1994). Thus, customers feeling guilty are less likely to bad mouth (Soscia, 2007) and are more likely to take reparative actions such as repurchase (Dahl et al., 2005). However, guilt is also associated with learning lessons, changing subsequent behavior, and avoiding the victim to avoid the reviving of unpleasant feelings of guilt (Baumeister et al., 1994). Therefore, customers who feel guilty also can have intentions to reduce future purchase (Burnett & Lunsford, 1994) or to switch to ethical alternatives in the future (Antonetti & Maklan, 2014).

#### Sickness Presence as a Trigger for Customer Compassion

Employee sickness presence may trigger compassion for the undeservingly suffering employee (goal incongruence, no agency of the employee) in customers and could also explain the negative effects on customer repurchase and recommendation intentions. Compassion arises from the appraisal of unfairness of another person’s suffering and the appraisal that this person cannot control or is responsible for the situation (Goetz et al., 2010). Feelings of compassion go along with actions (tendencies) that aim to reduce the suffering and reestablish justice, especially when one has the resources to help (Pfattheicher et al., 2019). In this regard, it has been shown that compassion can lead to prosocial behaviors (Batson & Shaw, 1991; Haid, 2009) and may lead to intentions to help the employee by repurchasing and recommending the service. In contrast, if the suffering is caused by an unjust action of a third entity, compassion can increase tendencies to punish this entity (Pfattheicher et al., 2019). In this process, compassion can evoke feelings of anger and, therefore, plays an indirect role in decisions to retaliate against perpetrators, for instance, in cases of corporate irresponsibility (Antonetti & Maklan, 2017). Thus, compassion might also reduce repurchase and recommendation intentions to sanction the company or management, if they are held responsible for employees suffering by the customers. In summary, the organizational consequences of compassion for repurchase and recommendation intentions are equivocal.

All mechanisms discussed are characterized by specific feelings, which can be empirically tested. These reactions are disgust and fear for disease avoidance, anger for personal anger and moral outrage, guilt for post-consumption guilt, and compassion for customer compassion for the employee (see Fig. 1). Thus, based on our reasoning about these mechanisms, we propose the following hypotheses for Study 3.

- *Hypotheses 2–4: *Customer disgust (Hypothesis 2), fear (Hypothesis 3), and anger (Hypothesis 4) mediate the negative effects of employee sickness presence on customer (a) repurchase intention and (b) recommendation intention.

The organizational consequences of costumer guilt and compassion are theoretically and empirically equivocal. Therefore, in Study 3, we explore the role of these affective mechanisms in explaining customer repurchase and recommendation intentions using the following research questions instead of proposing hypotheses.

- *Research Question 2 and 3:* Do costumer guilt (Research Question 2) and compassion toward the employee (Research Question 3) mediate the negative effects of service employee sickness presence on customer (a) repurchase intention and (b) recommendation intention?

## Study 3

### Method

We again used the experimental vignette methodology (Aguinis & Bradley, 2014) to test our hypotheses and to answer our research questions. As the statistical power of Study 3 was questioned during the review process, we conducted another study (Study 4) and describe the methods and results of Study 3 in abbreviated form. For reasons of transparency, however, we describe Study 3 in the Supplementary information in detail.

The vignettes we used (Table 1) were validated in an independent pilot study (*N* = 11 participants recruited through personal contacts). Overall, the results of the pilot study suggest that the two scenarios were sufficiently clear and distinct. Thus, we used the same scenarios in the main study. Detailed information is provided in Table S6 in the Supplementary information.

#### Participants and Procedure

Participants were recruited via announcements in a German university, recruited through requests via social networks and recruiting platforms, and through personal contacts. The online questionnaire was completed by 79 participants. We excluded one participant below the age of 18 years and six participants because they had missing data on outcome variables. The final sample consisted of *N* = 72 participants, including 49 women (68.1%), 17 men (23.6%), and one person indicating their gender as diverse (1.3%). Five persons (6.9%) did not indicate their gender. The average age of participants was 30.81 years (*SD* = 11.72) and ranged from 19 to 73 years. The participants were mostly employed (41.8%) or trainees and students (47.2%; Table S7). The procedure was as described in Study 1.

### Measures

#### Customer Repurchase and Recommendation Intentions

We used the same items as in Study 1 to examine customer repurchase and recommendation intentions. The correlation between these two items was *r* = 0.76 (*p* < 0.001) and, overall, the pattern of results was not substantially different to the results reported below when we used an average score of the two items in additional analyses.

#### Customers’ Affective Reactions

To measure customers’ affective reactions of disgust, fear, anger, and guilt during the service encounter, we adapted three translated items of the modified Differential Emotions Scale (Fredrickson, 2013) to assess each construct. These items are disgust, distaste, and revulsion for disgust; scared, fearful, and afraid for fear; angry, rage, and indignation for anger; and guilty, repentance, and blameworthy for guilt. Participants rated the strength of these affective reactions during the service encounter on a 7-point scale ranging from 1 (*not at all*) to 7 (*extremely*). The reliabilities of the scales were good (αs > 0.81). Compassion was measured with three adapted items from Landmann and Hess (2017). An example item is “I felt sorry for the courier.” The reliability of the scale was good (*α* = 0.88).

#### Customers’ Appraisals

We also assessed customers’ perceptions of fairness to examine customers’ appraisal of the service encounter. Appraisals are important for the differentiation of affective reactions (Moors et al., 2013), as the same feelings can have different causes and action tendencies. As we discussed personal anger and moral outrage as mechanisms that are both characterized by feelings of anger, customers’ appraisal of the situational unfairness and its target (i.e., customers themselves or the employee) could help to differentiate between the two mechanisms. For this purpose, we adapted three items from a distributive justice measure (Smith et al., 1999; e.g., “I got what I deserved”) to assess *perceptions of fairness toward the costumer* (personal fairness). Participants rated the items on a 7 − point scale ranging from 1 (*not at all true*) to 7 (*completely true*). The reliability of the scale was good (*α* = 0.89).

*Perceptions of company’s fairness toward its employee* (moral fairness) were measured with three items adapted from a fairness measure by Antonetti and Maklan (2016; e.g., “The company *Star Express* treats its employees in an unfair way.”). Participants rated the items on a 7-point scale ranging from 1 (*not at all true*) to 7 (*completely true*). Reliability was good (*α* = 0.92).

#### Statistical Analyses

We tested our hypotheses using the SPSS macro PROCESS (Hayes, 2018). The effects of employee sickness presence on customer repurchase and recommendation intentions and the mediating effects of disgust, fear, anger, guilt, and compassion were examined using regression analyses. We used heteroscedasticity consistent standard errors and covariance matrix estimators (Type HC3; Hayes & Cai, 2007) for all effects, except the indirect effects. Indirect effects were estimated with a bootstrapping procedure (Hayes, 2018) using a bootstrap sample size of 5000. The significance of the effects was tested at the 95% significance level. In additional analysis, we also used regression analysis to examine the effects of employee sickness presence on customers’ appraisals and the effects of customers’ appraisals on customer repurchase and recommendation intentions.

### Results and Discussion

Descriptive statistics and correlations of the study variables are shown in Table 5. An overview of the main results can be seen in Table 2.

**Table 5:**
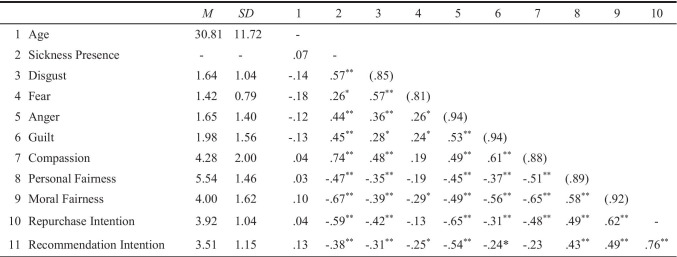
Descriptive statistics, reliabilities, and correlations between the variables (Study 3)

*N* = 67–72. Reliabilities (Cronbach’s *α*), where available, are reported in parentheses along the diagonal. Sickness Presence: not present (0), present (1)

^*^ *p* < .05; ** *p* < .01

In line with Hypotheses 1a and 1b, we replicated the negative effects of employee sickness presence on customer repurchase intention and recommendation intention (Table S8) that were also found in Study 1. Hypotheses 2 to 4 postulated that customers’ affective reactions of disgust, fear, and anger mediate the negative effects of employee sickness presence on customer repurchase and recommendation intentions, whereas Research Questions 2 and 3 explore potential mediations via guilt and compassion. As shown in Table S8, sickness presence had positive and significant effects on disgust, fear, anger, guilt, and compassion. However, as shown in Tables S9 and S10, only anger was negatively related to customer repurchase and recommendation intentions above and beyond the other affective reactions. Additionally, only anger mediated the negative effect of employee sickness presence on customer repurchase (indirect effect =  − 0.51, *SE* = 0.17, 95% CI =  − 0.87, − 0.20) and recommendation intentions (indirect effect =  − 0.53, *SE* = 0.17, 95% CI =  − 0.91, − 0.21). Therefore, Hypotheses 4a and 4b were supported.

There were no significant indirect effects of employee sickness presence on customer repurchase and recommendation intentions via disgust and fear (see Tables S9 and S10). Therefore, Hypotheses 2 and 3 were not supported. In addition, customer guilt and compassion did not mediate the negative effects of employee sickness presence on customer repurchase and recommendation intentions (Research Questions 2 and 3).

#### Additional Analysis

We also explored the effects of employee sickness presence on customers’ appraisals of the service encounter. As shown in Table S8, employee sickness presence had negative and significant effects on customers’ appraisals of personal fairness and moral fairness. In turn, only moral fairness had positive relationships with customer repurchase and recommendation intentions (Table S11).

Overall, in line with affective events theory (Weiss & Cropanzano, 1996), our results highlight the importance of customers’ affective reactions to sickness presence in developing their repurchase and recommendation intentions. Sickness presence evoked costumer disgust, fear, anger, guilt, and compassion. Therefore, we constructively replicated the findings of Study 2 using a quantitative approach. In addition, costumer feelings of anger mediated the negative effects of employee sickness presence on repurchase and recommendation intentions. Previous research found a comparable effect of sickness presence caused by employee mental illness, specifically feelings of depersonalization (Nesher Shoshan & Sonnentag, 2019). Surprisingly, no other affective reaction explained the detrimental effects of sickness presence on repurchase and recommendation intentions while accounting for the influence of the other affective reactions. This might be due to the specificity of behavioral intentions of the different affective reactions (Moors et al., 2013). Research suggests that anger is associated with confrontative coping strategies, such as intentions to harm wrongdoers (Yi & Baumgartner, 2004). In contrast, the other affective reactions might have evoked other cognitive strategies that help to cope with the unique situation. Another explanation might be the relatively small sample size of Study 3 that could limit the statistical power to detect further mechanisms (Fritz & MacKinnon, 2007).

In line with appraisal theories (Moors et al., 2013), employee sickness presence also elicited appraisals about the service encounter. Results show that sickness presence evoked perceptions of personal and moral (un)fairness. This is in line with research showing that anger is evoked by perceived unfair treatment of oneself but also others (Antonetti & Maklan, 2016). These results are the first evidence for customers’ personal anger and moral outrage in response to employee sickness presence. Additionally, moral fairness was related to costumer repurchase and recommendation intentions. Thus, customers’ moral outrage may be important in explaining reduced repurchase and recommendation intentions as response to employee sickness presence. This has important (practical) implications, as different types of anger require different forms of organizational actions, such as apologies, explanation, or compensation (Antonetti, 2016).

Additionally, the generalizability of the results to other types of service encounters could be questioned. Service encounters differ in duration (brief to extended), affective content (low to high arousal), and proximity of contact between service provider and customer (social/public to intimate/personal distance) and, therefore, have context-specific characteristics that can affect customers’ affective responses (Price et al., 1995). Due to the COVID-19 pandemic that began in early 2020, avoiding face-to-face contact (i.e., physical presence) and providing options for mediated communication (i.e., physical absence) became the most important factors to lower the risk of contagion (e.g., Rudolph et al., 2021).

Physical presence/absence of the customers may also be important for customer repurchase and recommendation intentions in service encounters with an ill employee. Cues of physical presence/absence influence individuals’ thoughts, affective reactions, and behavior (Henderson et al., 2011; Theodorakis & Painesis, 2018) because of the evolutionary hard-wired association between distance and safety (Williams & Bargh, 2008). Information of events becomes less salient if individuals are not present at the place where the event occurs and, therefore, individuals increasingly rely on assumptions or heuristics for evaluations. In addition, events elicit less emotional responding when individuals are physically absent (Hailey, 2014), and reduce sensitivity to emotion-laden attributes and emotional attachment to other persons (Williams & Bargh, 2008). It is shown that physical absence reduces unfavorable attitudinal (e.g., attitude toward brand) and behavioral (e.g., purchase intention) reactions of consumers to moral transgressions (Theodorakis & Painesis, 2018).

In summary, customers’ physical absence in a service encounter provides a lower risk of contagion and may reduce appraisals of unfairness and negative affective reactions, which might buffer detrimental effects on repurchase and recommendation intentions. Therefore, we propose that the indirect effects of employee sickness presence on customer repurchase and recommendation intentions (through the affective reactions) are weaker when customers are physically absent compared to when customers are physically present in a service encounter.

- *Hypothesis 5*: Physical presence/absence of customers in a service encounter moderates the positive effect of employee sickness presence on customer (a) disgust, (b) fear, (c) anger, (d) guilt, and (e) compassion, such that the effect is weaker when customers are physically absent compared to that when customers are physically present in the service encounter.

## Study 4

### Method

We again used the experimental vignette methodology (Aguinis & Bradley, 2014) to test our hypotheses and to answer our research questions. The vignettes we used (Table 1) were validated in an independent pilot study (*N* = 34 participants recruited through personal contacts). Results of the pilot study suggested that the scenarios were sufficiently clear and distinct, and the service encounters differ in terms of participants’ perceptions of physical distance, but not duration and affective arousal. Thus, we used the same scenarios in the main study. Detailed information is provided in Table S12 in the online supplemental material.

#### Participants and Procedure

We commissioned a certified panel management and online research company to recruit participants for this study. To ensure sample quality, the company recruits its participants using a variety of sources, from online communities and news portals to members-get-members campaigns, social media campaigns, and invitations after in-person interviews. All panelists register triple-opt-in and are deemed active according to ISO standards.

For an a-priori power analysis, we conducted a Monte Carlo study (Thoemmes et al., 2010) using Mplus (Muthén & Muthén, 1998–2015) to examine which sample size is needed to achieve a power of 0.80 given an alpha of 0.05 for our mediation model. Based on the effect sizes of Study 3, results showed that a total sample of 700 participants was required to achieve a power of 0.80 for all mediation effects.

The questionnaire was completed by *N* = 763 participants, including 384 men (50.3%), 376 women (49.3%), and three persons who did not indicate their gender (0.4%). The average age of participants was 46.01 years (*SD* = 15.02) and ranged from 18 to 74 years. About two-thirds of participants (65.3%) indicated to be employed or self-employed. They worked in a variety of industries, including commercial services, logistic and transport, and medicine and civil services. The other participants were retired (18.8%), trainees or students (8.7%), or unemployed (7.3%).

The procedure was as described in Study 1. Scenario 3a (no sickness presence, physical presence) was rated by 196 participants. Scenario 3b (sickness presence, physical presence) was rated by 193 participants, while 187 participants each rated Scenario 4a (no sickness presence, physical absence) and Scenario 4b (sickness presence, physical absence). The four groups were demographically very similar (Table S13).

### Measures

#### Customer Repurchase and Recommendation Intentions

We used three items each to measure customer repurchase and recommendation intentions (Zhang & Bloemer, 2008). Examples are “I consider the MARO bank as my first choice for banks” and “I say positive things about the MARO bank to other people.” Participants responded on 7-point scales ranging from 1 (*very unlikely*) to 7 (*very likely*). The reliabilities of the scales were good (αs > 0.93).

#### Customers’ Affective Reactions

We used the same items to measure customers’ affective reactions of disgust, fear, anger, guilt, and compassion during the service encounter as in Study 3. The reliabilities of the scales were good (αs > 0.92).

#### Customers’ Appraisals

To measure customers’ appraisals of goal congruence, agency, situational control, and certainty during the interaction between them and the bank employee, we adapted items from previous studies (Hosany, 2012; Ruth et al., 2002). Participants responded on 7-point scales ranging from 1 (*not at all*) to 7 (*very much*). *Goal congruence* was measured with five items asking about goal relevance, goal-related valence, and goal consistency. An example is “By talking to the bank employee, I achieved what I wanted to achieve.” *Agency* of the bank, the management, and the customer was measured with two items each. An example for agency of the bank employee is “The bank employee is responsible for the circumstances under which the conversation took place.” *Situational control* was measured with two items, “The circumstances were beyond anyone’s control” and “The circumstances were mere coincidence.” *Certainty* was measured with a single item, “I was sure about what happened in the conversation with the bank employee.” Reliabilities were good (αs > 0.70).

#### Statistical Analyses

We tested our hypotheses as described in Study 3. In addition, we estimated conditional indirect effects with a bootstrapping procedure (Hayes, 2018) using a bootstrap sample size of 1000.

### Results and Discussion

Descriptive statistics and correlations of the study variables are shown in Table 6.

**Table 6:**
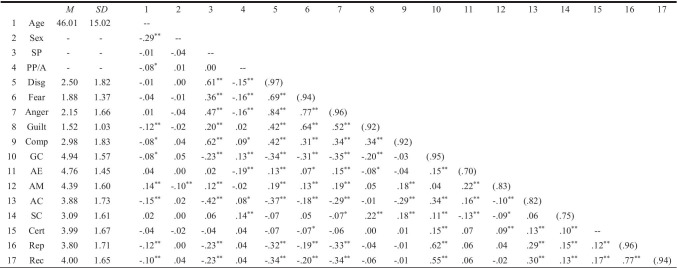
Descriptive statistics, reliabilities, and correlations between the variables (Study 4)

*N* = 753–763. Reliabilities (Cronbach’s *α*), where available, are reported in parentheses along the diagonal. Sex: male (0), female (1); *SP*, Sickness Presence: not present (0), present (1); *PP/A*, Physical Presence/Absence of the customer during the service encounter: presence (0), absence (1); *Disg*, Disgust; *Comp*, Compassion; *GC*, Goal Congruence; *AE*, Agency Employee, *AM*, Agency Management; *AC*, Agency Customer; *SC*, Situational Control; *Cert*, Certainty; *Rep*, Repurchase Intention; *Rec*, Recommendation Intention

^*^ *p* < .05; ** *p* < .01

Replicating the results of Studies 1 and 3, and consistent with Hypothesis 1, there were negative effects of employee sickness presence on customer repurchase and recommendation intentions (Table 7). Additionally, results showed that sickness presence and costumers’ physical presence/absence positively interacted in predicting recommendation intention. The conditional direct effect of employee sickness presence on customer recommendation intention was weaker when customers were physically absent compared to physically present.

**Table 7:** Direct effects and interaction effects of employee sickness presence and customers’ physical presence/absence on the mediators and the dependent variables (Study 4)

	Disgust (*M1*) *B* (*SE*)	Fear (*M2)* *B (SE)*	Anger (*M3*) *B* (*SE*)	Guilt (*M4*) *B* (*SE*)	Compassion *(M5)* *B* (*SE*)	Repurchase Intention *B (SE)*	Recommendation Intention *B (SE)*
Sickness Presence (*X*)	2.76 (0.14)**	1.44 (0.13)**	2.08 (0.16)**	0.48 (0.10)**	1.88 (0.15)**	− 0.95 (0.17)**	− 1.15 (0.16)**
Physical Presence/Absence (*Mod*)	− 0.02 (0.10)	0.02 (0.09)	− 0.02 (0.10)	0.11 (0.09)	− 0.05 (0.13)	− 0.04 (0.17)	− 0.24 (0.17)
X*Mod	− 1.09 (0.20)**	− 0.94 (0.18)**	− 1.05 (0.21)**	− 0.14 (0.15)	0.77 (0.21)**	0.37 (0.24)	0.76 (0.23)**
Constant	1.40 (0.07)**	1.39 (0.06)**	1.38 (0.07)**	1.26 (0.05)**	1.88 (0.09)**	4.20 (0.12)**	4.50 (0.11)**
*R*^*2*^	.42**	.18*	.27**	.04**	.40**	.06**	.07**

*N* = 763. Sickness Presence: not present (0), present (1); Physical Presence/Absence of the customer during the service encounter: presence (0), absence (1); Unstandardized regression coefficients and robust standard errors are reported. *p < .05; ** *p* < .01

Hypotheses 2 to 4 postulated that customers’ affective reactions of disgust, fear, and anger mediate the negative effects of employee sickness presence on customer repurchase and recommendation intentions, whereas Research Questions 2 and 3 explore potential mediations via guilt and compassion. As shown in Table 7, sickness presence had positive and significant effects on disgust, fear, anger, guilt, and compassion, which replicates the findings of Study 3. Again, anger was negatively related to *customer repurchase intention* (Table 8). In addition, we found that disgust was negatively and guilt and compassion were positively related to customer repurchase intention. Therefore, disgust (indirect effect =  − 0.27, *SE* = 0.14, 95% CI =  − 0.54, − 0.01) and anger (indirect effect =  − 0.55, *SE* = 0.11, 95% CI =  − 0.77, − 0.34) mediated negative effects of employee sickness presence on customer repurchase intentions, whereas guilt (indirect effect = 0.07, *SE* = 0.03, 95% CI = 0.01, 0.13) and compassion (indirect effect = 0.41, *SE* = 0.10, 95% CI = 0.22, 0.61) mediated positive effects (Table S14). There was no indirect effect via fear. The total effect of sickness presence on customer repurchase intentions was negative and significant (*B* =  − 0.77, *SE* = 0.12, *p* < 0.001). Therefore, Hypothesis 2a and again Hypothesis 4a were supported, whereas Hypothesis 3a was, as in Study 3, not supported.

**Table 8:** Direct and conditional indirect effects of employee sickness presence on repurchase and recommendation intentions (Study 4)

		Repurchase Intention			Recommendation Intention	
		*B* (*SE*)			*B* (*SE*)	
Sickness Presence (*X*)		− 0.56 (0.17)**			− 0.57 (0.17)**	
Disgust (*M1*)		− 0.12 (0.06)*			− 0.15 (0.07)*	
Fear (*M2*)		0.13 (0.07)			0.14 (0.06)*	
Anger (*M3*)		− 0.35 (0.07)**			− 0.31 (0.07)**	
Guilt (*M4*)		0.16 (0.07)*			0.10 (0.07)	
Compassion (*M5*)		0.18 (0.05)**			0.19 (0.04)**	
Constant		4.11 (0.14)**			4.34 (0.13)**	
*R*^2^		.17**			.17**	
Conditional indirect effects of *X* on the outcomes at two conditions of customers’ Physical Presence/Absence						
			Repurchase intention		Recommendation intention	
Mediator	Physical Presence/Absence		*B* (*SE*)	95% CI	*B* (*SE*)	95% CI
Disgust	Presence (0)		− 0.34 (0.18)*	− 0.69; − 0.00	− 0.41 (0.18)*	− 0.77; − 0.09
	Absence (1)		− 0.20 (0.11)*	− 0.43; − 0.00	− 0.25 (0.11)*	− 0.46; − 0.05
Fear	Presence (0)		0.19 (0.10)	− 0.02; 0.38	0.20 (0.10)*	0.04; 0.39
	Absence (1)		0.07 (0.04)*	0.00; 0.16	0.07 (0.04)*	0.01; 0.16
Anger	Presence (0)		− 0.73 (0.15)*	− 1.05; − 0.47	− 0.64 (0.14)*	− 0.96; − 0.40
	Absence (1)		− 0.36 (0.08)*	− 0.56; − 0.22	− 0.32 (0.08)*	− 0.49; − 0.19
Guilt	Presence (0)		0.08 (0.04)*	0.01; 0.17	0.05 (0.03)	− 0.02; 0.12
	Absence (1)		0.05 (0.03)*	0.00; 0.13	0.03 (0.03)	− 0.01; 0.10
Compassion	Presence (0)		0.34 (0.09)*	0.17; 0.53	0.36 (0.08)*	0.20; 0.53
	Absence (1)		0.48 (0.12)*	0.23; 0.72	0.51 (0.11)*	0.30; 0.74
Index of moderated mediation						
			Repurchase intention		Recommendation intention	
Mediator			Index (*SE*)	95% CI	Index (*SE*)	95% CI
Disgust			0.13 (0.07)*	0.01; 0.29	0.16 (0.08)*	0.04; 0.34
Fear			− 0.12 (0.07)	− 0.28; 0.00	− 0.13 (0.06)*	− 0.28; − 0.03
Anger			0.37 (0.10)*	0.21; 0.61	0.32 (0.10)*	0.17; 0.55
Guilt			− 0.02 (0.03)	− 0.11; 0.01	− 0.01 (0.02)	− 0.08; 0.01
Compassion			0.14 (0.05)*	0.06; 0.26	0.15 (0.05)*	0.06; 0.26

*N* = 763. Sickness Presence: not present (0), present (1); *CI*, confidence interval; Unstandardized regression coefficients and robust standard errors are reported. Bootstrap sample size = 1000; The index of moderated mediation is a test of equality of the conditional indirect effects in the two groups

^*^p < .05; **p < .01

Anger was negatively related to *customer recommendation intention*, which is replicating the findings of Study 3. We also found that disgust was negatively and fear and compassion were positively related to customer recommendation intention (Table 8). Additionally, disgust (indirect effect =  − 0.33, *SE* = 0.14, 95% CI =  − 0.63, − 0.08) and anger (indirect effect =  − 0.49, *SE* = 0.10, 95% CI =  − 0.68, − 0.27) mediated negative effects of employee sickness presence on customer recommendation intentions, whereas fear (indirect effect = 0.13, *SE* = 0.06, 95% CI = 0.02, 0.25) and compassion (indirect effect = 0.44, *SE* = 0.09, 95% CI = 0.25, 0.63) mediated positive effects (Table S15). There was no indirect effect via guilt. The total effect of sickness presence on customer recommendation intentions was negative and significant (*B* =  − 0.77, *SE* = 0.12, *p* < 0.001). Therefore, Hypotheses 2b and 3b and again Hypothesis 4b were supported.

Hypothesis 5 postulated that costumers’ physical presence/absence moderates the positive effect of employee sickness presence on customer (a) disgust, (b) fear, (c) anger, (d) guilt, and (e) compassion, such that the effect is weaker when customers are physically absent compared to that when customers are physically present. Results showed that employee sickness presence and customers’ physical presence/absence interacted in predicting disgust, fear, anger, and compassion (Table 7). The positive effect of sickness presence on disgust, fear, and anger was weaker when customers were absent (compared to present), whereas the positive effect of sickness presence on compassion was stronger when customers were absent (compared to the present). Therefore, Hypotheses 5a to 5c were supported, whereas 5d and 5e were not supported.

**Fig. 2:**
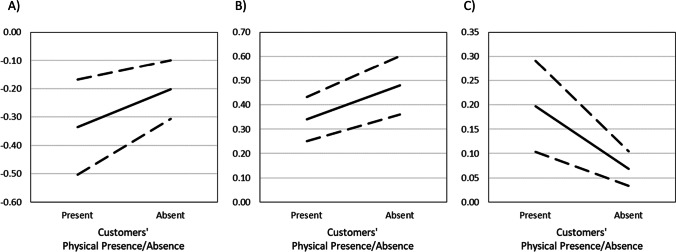
Results of Study 4. Plots show the conditional indirect effects of employee sickness presence on repurchase intentions through (**A**) disgust and (**B**) compassion, and (**C**) the conditional indirect effect of employee sickness presence on recommendation intention through fear, with their continuous lower (− 1 *SE*) and upper bounds (+ 1 *SE*; in dashed lines) at two conditions of customers’ physical presence/absence during the service encounter

The indirect effect of employee sickness presence on *customer repurchase intention* through disgust, anger, and compassion was conditional on the physical presence/absence of the customer (Table 8). The negative indirect effects via disgust and anger were weaker if the customers were absent (compared to present), whereas the positive indirect effect via compassion was stronger if the customer were absent (compared to the present; see Fig. 2). The indirect effect via guilt did not depend on physical presence/absence. The indirect effect of employee sickness presence on *customer recommendation intention* through disgust, fear, anger, and compassion depended on the physical presence/absence of the customer (Table 8). The negative indirect effects via disgust and anger were weaker if the customers were absent (compared to the present). The positive indirect effect via fear was weaker if the customers were absent (compared to the present; see Fig. 2), whereas the positive indirect effect via compassion was stronger if the customers were absent (compared to the present).

#### Additional Analyses

We also explored the effects of employee sickness presence on customers’ appraisals of the service encounter. As shown in Table S16, employee sickness presence had negative and significant effects on customers’ appraisals of goal congruence, agency of the customer, and certainty, whereas employee sickness presence had a positive and significant effect on agency of the management. Additionally, results showed that employee sickness presence and physical presence/absence of the customer positively interacted in predicting customers’ appraisals of goal congruence and certainty. The negative effect of employee sickness presence on customers’ appraisals of goal congruence was weaker when the customers were absent (compared to the present). There was only a negative effect of employee sickness presence on customers’ appraisals of certainty when the customers were present, but there was no effect when the customers were absent. In addition, customers’ appraisals of goal congruence, agency of the customer, and situational control had positive relationships with customer repurchase intention (Table S17). Customer recommendation intention was positively related to goal congruence, agency of the customer, situational control, and certainty.

Overall, the results of Study 4 show that a service encounter with an ill employee elicits customers’ appraisals and affective reactions and that these reactions are important in the development of customer repurchase and recommendation intentions. This is in line with affective events theory (Weiss & Cropanzano, 1996) and appraisal theories (Moors et al., 2013), and it replicates the findings of Study 2 and Study 3 (see Table 2). Specifically, customers confronted with an ill employee appraised the service as less congruent with their goal to open an account at the bank, assigned more control and responsibility for the service encounter to the management of the bank, and had less repurchase and recommendation intentions. Among other affective reactions, customers experienced anger, which reduced their repurchase and recommendation intentions. Thus, these results may provide evidence for the proposed mechanism personal anger. Customers appraise the thwarting of their own interests and react with anger, which might be directed toward the management rather than the employee as the management is appraised to have more agency.

Furthermore, disgust explained the negative effect of employee sickness presence on both customer intentions. Disgust should be accompanied by appraisals of goal incongruence and agency of circumstances (Ma et al., 2013). Nevertheless, employee sickness presence had no effect on appraisals about situational control. Therefore, customers’ response to employees’ illness might be not completely conscious, which is in line with research on disease avoidance (Park et al., 2003). In summary, these results provide first evidence for a disease avoidance mechanism characterized by feelings of disgust. Disease avoidance can also be characterized by fear and uncertainty. Indeed, employee sickness presence evoked fear in customers and appraisals of uncertainty. Nevertheless, fear did not explain the negative effect of employee sickness presence on customer repurchase intention. Customers feeling scared, afraid, or uncertain showed even more recommendation intentions. This is contrary to our assumption that fear goes along with negative attitudes and a tendency to distance from the eliciting stimulus (Öhmann, 2008). Maybe this tendency relates only to the current encounter but not to future actions. In addition, recommendation of a service is a low risk behavior for the customer and the increased intentions might reflect customers’ attempts to justify their decision for the service provider, increase their self-esteem in an uncertain situation, or reduce cognitive dissonance to have purchased the service (Keiningham et al., 2018). Thus, talking about the service might be a strategy to handle uncertainty for customers being particularly scared but not disgusted. This might reflect another mechanism mostly characterized by fear, which, however, cannot explain the negative effects of sickness presence on repurchase and recommendation intentions.

Exploring the explanatory value of guilt, we found that this affective reaction in response to sickness presence can increase customer repurchase intention. Previous research showed that post-consumption guilt can increase intentions for specific reparative actions toward salespersons with whom customers felt socially connected (Dahl et al., 2005). Consistently, our results show that feeling guilty may increase tendencies for general reparative actions like repurchase. In addition, feelings of guilt should be accompanied by appraisals of self-agency (Ma et al., 2013). Nevertheless, sickness presence reduced appraisals of self-agency in customers. In summary, feelings of guilt cannot explain the negative effect of sickness presence on customer intentions. As sickness presence reduces appraisals of self-agency in customers, post-consumption guilt may play only a minor role in explaining customers’ reactions to employee sickness presence.

Customers’ feelings of compassion in response to employee sickness presence were positively associated with customer repurchase and recommendation intentions. In addition, employee sickness presence related positively to customers’ appraisals of responsibility and control by the management, while it was not associated with appraisals about employees’ agency. This might indicate that customers hold unjust actions by the management particularly responsible for employees’ suffering but not the employees themselves. These customers may also experience anger and have increased tendencies to punish the management, which can be seen as form of moral outrage (Pfattheicher et al., 2019). Such a process would explain the negative effects of employee sickness presence on customer intentions. However, customers focusing on the low responsibility and control of the employees might mostly feel compassion and have intentions to help the (undeservingly suffering) employee. Thus, their increased repurchase intentions might reflect intentions for prosocial behavior (Pfattheicher et al., 2019), whereas recommendation intentions could be a form of prosocial lying about the service experience (Lupoli et al., 2017). In summary, compassion without anger cannot explain the negative effect of employee sickness presence on customer intentions.

Physical presence/absence of the customers in the service encounter appears to be an important characteristic of service encounters to reduce the risk of infection and the detrimental impact of employee sickness presence on customer intentions. Physical absence mitigates the eliciting of customers’ disgust, fear, and anger, as well as appraisals of goal incongruence and uncertainty in response to employee sickness presence. This could be explained by higher perceived safety (Williams & Bargh, 2008) and less salient details of the service encounter (Henderson et al., 2011). At the same time, feelings of compassion due to employee sickness presence are exacerbated in customers calling the hotline compared to customers going to the branch. This is contrary to our expectation that physical absence reduces emotional responding (Hailey, 2014) and emotional attachment to other persons (Williams & Bargh, 2008). A possible explanation could be that physically absent customers rely on assumptions and heuristics to evaluate the service encounter rather than on actual details (Henderson et al., 2011). In this case, customers’ assumptions about the specific services might have a strong influence. For example, a customer could have other expectations about the working conditions of employees responding to phone calls compared to employees working in a branch of a bank. Indeed, we found that customers ascribed less agency to the employee responding to the call compared to the employee in the branch. In consequence, customer compassion might be exacerbated for employees responding to phone calls as customers expect that these employees have less control over and are less responsible for the service encounter inclusive their bad health conditions.

## General Discussion

With this paper, we contribute to a better understanding of the effects of employee sickness presence on other stakeholders (i.e., customers) than employees themselves. In line with affective events theory (Weiss & Cropanzano, 1996), results of our studies broadly overlapped in suggesting that an interaction with an ill employee is an affective event and elicits various affective reactions in customers, including mostly affective reactions with a negative valence, but also ambiguous affective reactions. These affective reactions are disgust, fear, anger, guilt, and compassion. Furthermore, the results of our studies suggest an explanatory value of these affective reactions for the effect of sickness presence on customer intentions, although there were some divergent findings in Studies 3 and 4 (Table 2). Anger explains a negative effect of employee sickness presence on customer repurchase and recommendation intentions in both studies, while an explanatory value of disgust for this negative effect was solely found in Study 4. In addition, in Study 4, fear explained a positive effect of employee sickness presence on customer recommendation intention, whereas guilt explained a positive effect on repurchase intention. Compassion mediated positive effects of employee sickness presence on both intentions in Study 4. Overall, employee sickness presence reduces customer repurchase and recommendation intention even if the positive indirect effects are accounted for.

In line with appraisal theories (Moors et al., 2013), we also found evidence for customers’ appraisals as a response to employee sickness presence. These appraisals are personal and moral unfairness as well as goal incongruence, agency of the management, reduced agency of the customer, and uncertainty. Moral unfairness and goal incongruence were negatively associated with customer repurchase and recommendation intentions, whereas agency of the customer was positively associated with both customer intentions. Certainty was positively associated with recommendation intentions. Thus, we found evidence that disease avoidance, personal anger, and moral outrage could be central mechanisms underlying the negative effects of employee sickness presence. Furthermore, we found evidence that fear, post-consumption guilt, and compassion could be central mechanisms underlying positive effects of employee sickness presence on customer intentions. In summary, we show the generalizability of the negative effects of employees’ physical illness on customer return and recommendation intentions (Correia Leal &Ferreira, 2019) to the courier, express, and parcel industry as well as banking services. Additionally, we expanded current knowledge by examining the underlying mechanisms.

In cases of unavoidable employee sickness presence, providing options for physical absence of customers during service encounters (e.g., consulting via phone) may be useful to reduce the risk of contagion. It may be also a strategy to maintain customer repurchase and recommendation intentions as physical absence mitigates feelings of disgust and anger, as well as increased compassion in response to employee sickness presence. However, physical absence also minimizes feelings of fear, which comes at the costs of lowered recommendation intentions.

### Limitations and Future Research

Our study has some limitations that should be considered when interpreting the results. First, there might be a lack of generalizability due to the experimental designs of Studies 1, 3, and 4. Experimental vignette designs with “paper people” can be criticized for providing only very few cues and little contextual information for participants to react upon. We attempted to reduce concerns about external validity by carefully constructing realistic scenarios (Aguinis & Bradley, 2014). At least package deliveries are representative for daily service encounters (Mattila & Enz, 2002). Additionally, we examined customers’ real-life experiences (Study 2) to ensure that customers remembered an actual service employee’s illness in their daily life. A promising direction for future studies would be to use other experimental approaches (e.g., actor performing sickness cues) or observational and survey-based field data. For instance, a field study could examine customer repurchase and recommendation intentions in relation to the average of sickness presence days of an employee or working team.

Second, the cross-sectional designs of Studies 3 and 4, particularly the associations between affective reactions, appraisals, and repurchase and recommendation intentions, do not allow causal interpretations. However, cross-sectional data are useful in exploratory research with a large set of potential causes related to the outcome and when the appropriate time frame is not known (Spector, 2019). Future studies could additionally manipulate the affective reactions and appraisals and examine their effects on repurchase and recommendation intentions. Additionally, the very low means of the affective reactions and the small sample size of Study 3 could be limitations regarding statistical power to detect the mechanisms of the effects of employee sickness presence on repurchase and recommendation intentions (Fritz & MacKinnon, 2007).

Third, the manipulation of sickness presence was relatively simple (i.e., present or absent), whereas the variable can be considered continuous with different qualitative characteristics (e.g., kind of sickness or symptoms). Future studies should examine other physical as well as mental diseases, their symptoms, and possible interactions of these symptoms. For example, symptoms of burnout seems to interact in predicting customers’ perceptions of employees’ and organizations’ services (Nesher Shoshan & Sonnentag, 2019). In addition, the validation of the scenarios used in Studies 1 and 3 could be affected by experimenter demands as the pilot studies were relatively short (reading the scenario and indicating whether the employee was sick or not). In Study 4, we used a between-person design, which may somewhat alleviate such concerns.

Finally, we cannot compare the risks of employee sickness presence for customer repurchase and recommendation intentions arising in other types of service encounters and for other kinds of symptoms. Previous studies showed detrimental effects of employee sickness presence due to a severe cold in a service encounter with physically present customers (front office in hospitality; Correia Leal & Ferreira, 2019). In Study 4, we systematically compared two scenarios differing in terms of physical presence/absence of the customers, holding constant duration and affective arousal. The two scenarios in Studies 1 and 3 differ particularly in terms of duration. Duration could moderate the detrimental effects of employee sickness presence as thoughtful processes and rule-based reactions of customers take some time to develop compared to spontaneous and impulsive reactions (Pryor et al., 2004). Service encounters with a longer duration and less personal distance provide opportunities for employees’ self-revelation and sharing of feelings (Price et al., 1995). However, extended and close service encounters also provide more opportunities for interferences due to obstacles and problems (Price et al., 1995) that might be caused by sickness (Schultz & Edington, 2007).

Thus, future studies could examine whether the effects and the underlying mechanisms of employee sickness presence differ depending on further characteristics of service encounters, such as duration (e.g., hair dressing vs. parcel delivery) or affective arousal (e.g., tattoo artist vs. nail care). In addition, different symptoms of illness may elicit different affective reactions or even interact in predicting customer intentions. For example, employees’ emotional exhaustion can mitigate the negative effect of their depersonalization on customer service perceptions via customer anger (Nesher Shoshan & Sonnentag, 2019). Customers recognizing employees’ exhaustion might have felt more compassionate than angry. Thus, it is a promising avenue to systematically examine combinations of different service encounters and symptoms of illness to uncover their interaction in predicting customer intentions.

### Practical Implications

Sickness presence is not only harmful for employee health (Lohaus & Habermann, 2019), but can also decrease repurchase and recommendation intentions and, therefore, may have negative consequences for business performance (Morgan & Rego, 2006). Thus, employees and companies should take sickness presence seriously. A first step may be to create health-promoting and maintaining working conditions as employees’ health status is one of the most important predictors for sickness presence (Lohaus & Habermann, 2019). For example, job insecurity and job demands, such as overtime or understaffing, are directly related to employees’ health and therefore, have a relationship with sickness presence (Miraglia & Johns, 2015). Such negative working conditions can also have an effect on sickness presence via the imposition of attendance pressure (Ruhle et al., 2019). However, practitioners should keep in mind that good working conditions, such as collegial and supervisor support, represent a double-edged sword as they promote employee health, but are also related to job satisfaction, which can positively influence employees’ decision to work while ill (Miraglia & Johns, 2015). Thus, as not all health impairments can be prevented, managers should send ill employees home whenever possible.

However, if sickness presence reflects a sustainable choice for the employee, it has to be properly managed. Sickness presence caused by non-contagious illness could be functional or therapeutic, when the working conditions allow the employee to work within the boundaries of their illness-reduced resources and the level of effort is not extreme (Karanika-Murray & Biron, 2019). For this purpose, meeting ill employees’ special needs, such as adjustable work quantity and quality, is crucial. A strategy could be to provide employees easy access to replacements or options for job rotation and, therefore, avoid contact with customers or at least provide options for the physical absence of the customers during the service encounter.

Service providers could also inform customers about the sustainable sides of their employees’ sickness presence and their effort to support ill employees to recover on the job. Customers valuing responsible treatment of employees may have reduced moral outrage reactions due to sickness presence and, therefore, less negative intentions toward the company, when they recognize such socially responsible behavior by the company (Joireman et al., 2015). In addition, customer compassion for the ill employee may mitigate intentions to harm the company and could increase repurchase and recommendation intentions. However, whenever the employee cannot maintain high service quality or sickness presence is detrimental for health, employers should encourage them to take sick leave.

## Conclusion

We examined the negative effects of service employee sickness presence on customer repurchase and recommendation intentions via customers’ affective reactions. In this process, customer disease avoidance, personal anger, and moral outrage seem to play a crucial role. In addition, customer compassion for the employee could be a promising mechanism to mitigate detrimental effects on customer repurchase and recommendation intentions if employee sickness presence is a sustainable choice for the employee. Providing options for the physical absence of the customers during the service encounter (e.g., communication via phone) reduces the risk of contagion and may be a strategy to maintain customer repurchase and recommendation intentions. The results of our studies provide knowledge about the risks of sickness presence for organizational stakeholders and stress the importance of preventing and sustainably managing sickness presence in the service industry.

## Supplementary Information

Below is the link to the electronic supplementary material.

Supplementary file1 (DOCX 103 KB)
