# Supplementary material for: Effects of Employee Sickness Presence on Customer Repurchase and Recommendation Intentions: The Role of Customer Affective Reactions
Source: J Bus Psychol. 2021 Aug 16;37(4):831–54. doi: 10.1007/s10869-021-09764-1 (PMC8366743; doi:10.1007/s10869-021-09764-1)
Supplement: Supplementary file 1 — Supplementary file1 (DOCX 103 KB) [file 10869_2021_9764_MOESM1_ESM.docx]

**Online supplemental material to accompany:**

(Blinded for review)

Table S1

*Results of the validation of the vignettes used in Study 1*

|  |  | *M* | *SD* | *t* | *p* |
| --- | --- | --- | --- | --- | --- |
| Health Impairments | Scenario 1a | 4.60 | 0.52 |  |  |
|  | Scenario 1b | 1.40 | 0.52 | -16.00 | < .001 |
| *Note*. *N* = 10. The sample included eight women and two men between 21 and 37 years (*M_age_* = 25.50, *SD*= 5.30). Participants were asked to rate the probability of the health impairment of the employee, who was described in the vignettes, on a 5‑point scale (1 = *very unlikely*, 5 = *very likely*) for each scenario. In one of the two scenarios, the customer has no information about the employee’s health status (Scenario 1a). In the other scenario, we added a cue about the employee’s health impairment (Scenario 1b) to manipulate employee sickness presence. | | | | | |

Table S2

*Demographic characteristics for two groups of participants separated by the rated scenario (Study 1)*

|  | Proportion of | |
| --- | --- | --- |
|  | participants rating Scenario 1a | participants rating Scenario 1b |
|  | in % | in % |
| Sex |  |  |
| Women | 68.3 | 64.4 |
| Men | 31.7 | 35.6 |
| Industry |  |  |
| Academia | 9.5 | 9.9 |
| Banking and insurance companies | 4.8 | 3.0 |
| Economics | 4.8 | 4.0 |
| Educational professions | 19.8 | 15.8 |
| Engineering | 10.3 | 6.9 |
| Hospitality | 0.8 | 2.0 |
| Logistic and transport | 2.4 | 3.0 |
| Medicine and civil service | 19.8 | 27.7 |
| Public administration | 8.7 | 10.8 |
| Without profession | 19.0 | 16.8 |
| *Note.* In Scenario 1a, the customer has no information about the employee’s health status. In Scenario 1b, we added a cue about the employee’s health impairment to manipulate employee sickness presence. The average age of participants rating Scenario 1a was 29.98 years (*SD* = 8.92). The average age of participants rating Scenario 1b was 28.34 years (*SD* = 8.40). | | |

| Table S3  *Test of ANOVA assumptions and effects of employee sickness presence on customer repurchase and recommendation intentions (Study 1)* | | | | | | | | | | | | | | | | | |
| --- | --- | --- | --- | --- | --- | --- | --- | --- | --- | --- | --- | --- | --- | --- | --- | --- | --- |
|  |  | Shapiro-Wilk test | | | Levene’s test | | | | | One-way analysis of variance | | | | | | | |
|  |  | *W* | *df* | *p* | *L* | *df1* | *df2* | *p* | *N* | | *M* | *SD* | *F* | *df1* | *df2* | *p* |  |
| Repurchase Intention | Scenario 1a | 0.78 | 126 | < .001 |  |  |  |  | 126 | | 4.18 | 0.92 |  |  |  |  |  |
|  | Scenario 1b | 0.89 | 101 | < .001 |  |  |  |  | 101 | | 3.50 | 1.21 |  |  |  |  |  |
|  | Total |  |  |  | 17.25 | 1 | 225 | < .001 | 227 | | 3.88 | 1.11 | 22.34 | 1 | 183.84 | < .001 |  |
| Recommen-dation Intention | Scenario 1a | 0.81 | 126 | < .001 |  |  |  |  | 126 | | 4.08 | 0.96 |  |  |  |  |  |
|  | Scenario 1b | 0.88 | 101 | < .001 |  |  |  |  | 101 | | 3.50 | 1.20 |  |  |  |  |  |
|  | Total |  |  |  | 14.47 | 1 | 225 | <.001 | 227 | | 3.82 | 1.11 | 15.35 | 1 | 189.44 | < .001 |  |
| *Note.* *L* = Levene’s statistic based on the mean; *F* = Welch’s *F.* | | | | | | | | | | | | | | | | | |

| Table S4  *Customers’ original and translated (affective) reactions and thoughts about sickness presence of service employees* | | | | | | | | |
| --- | --- | --- | --- | --- | --- | --- | --- | --- |
| No. | Original (affective) reactions | Translated (affective) reactions | R1 | R2 | Original thoughts | Translated thoughts | R1 | R2 |
| 5 | Als unangenehm und (bzgl. meiner eigenen Gesundheit sowie der anderer Gäste) bedrohlich. Und ich hab mich geärgert, dass die Kellnerin nicht einfach zu Hause geblieben ist und sich erholt hat. | As unpleasant and, with regard to my own health and the health of other guests, as threatening. And I was angry that the waitress did not simply stay at home and recovered. | 5  2  1 | 1  2  3 | Warum musst du mit einer Erkältung unbedingt arbeiten gehen? | Why do you have to go to work with a cold? | 4 | 4 |
| 6 | Mitleid, der arme | Compassion, the poor guy. | 6 | 6 | Das bringt die Weihnachtszeit eben so mit sich - evtl hat er sich ja auch als Saisonarbeiter eintragen lassen (war ein neuer Postbote) | That's normal during Christmas season - maybe he has registered as a seasonal worker (he was a new postman). | 2 | 3  8 |
| 15 | Ich fand es unangemessen, dass jemand der sichtbar krank ist, arbeiten muss. | I found it inappropriate that someone who is visibly ill has to work. | 5 | 8 | Er sollte sich lieber ausruhen, damit es sich nicht verschlimmert | He would be better off resting so that it does not get worse. | 6  7 |  |
| 16 | Ich hatte nicht das Gefühl, dass sie voll leistungsfähig war und habe nicht den Service bekommen, den ich mir erhofft hatte. | I did not feel that she was fully capable and I did not get the service I hoped for. | 5 | 3 | Sie tat mir leid, da sie sich sichtlich quälte. | I felt sorry for her, as she was visibly struggling. | 6  8 | 6  8 |
| 19 | gleichgültig, Dienstleistender tat mir leid, führte jedoch nicht zu Einschränkung der Dienstleistung | Indifferent, I felt sorry for the service provider, but it did not lead to a restriction of the service. | 8  6 | 8 | Verständnis gegenüber dem Dienstleistenden, schwer Schichten abzusagen | Understanding towards the service provider, difficult to cancel shifts. | 2 | 2 |
| 23 | eher störend aber durch die kürze auch schnell in Richtung gleichgültigkeit | Rather annoying but due to the short time it quickly lead to indifference. | 5  8 | 5 | Stress, Konkurrenzkampf, Müdigkeit der dienstleistenden Person, Erpressung (Druck), Niedriglohn | Stress, competition, fatigue of the person providing the service, blackmail (pressure), low wage. | 1  2  6 | 1 |
| 24 | Etwas unangenehm, weil ich gesund Zuhause saß und die Dienstleistende gehetzt versucht hat Pakete zuzustellen | Somewhat unpleasant, because I was sitting at home healthy and the service provider was hastily trying to deliver parcels. | 5 | 4 | Ich habe gehofft, dass sie bald fertig mit ihrer Post-Runde ist und bald nach Hause kann, um sich gesund zu schlafen | I was hoping that she would soon be finished with her mail delivery and could soon go home to get a good night's sleep. | 7 | 7 |
| *Continued* | | | | | | | | |
|  | | | | | | | | |
|  | | | | | | | | |
|  | | | | | | | | |
| Table S4 *Continued* | | | | | | | | |
| No | Original (affective) reactions | Translated (affective) reactions | R1 | R2 | Original thoughts | Translated thoughts | R1 | R2 |
| 26 | Ich war verunsichert, da ich bei einem neuen Friseur was und Angst hatte ob die Krankheit der Friseurin den Haarschnitt beeinflusst. | I was unsettled, because I visited a new hair stylist and was afraid that the illness of the stylist would influence the haircut. | 2 | 2 | Ansteckungsgefahr, unwohl dass sie meine Haare berührt | Risk of infection, uncomfortable that she touches my hair. | 5 | 5 |
| 29 | Nicht schön, Mitgefühl, Empörung über Firma | Not good, compassion, outrage about the company | 3  6 | 3  6 | Das System zwingt Menschen in die Knie, krank sollte man nicht arbeiten müssen, die Druck ist zu hoch, Scheiss LeistungsGesellschaft | The system forces people to their knees, sick people should not have to work, the pressure is too high, shitty achievement-oriented society. | 1 | 1 |
| 31 | Ich empfinde dies nicht als schlimm. Es wird nach Stunden bezahlt und ich muss nicht wegen jedes Schnupfens Zuhause bleiben. | I did not experience this as too bad. Wages are paid by the hour and I don’t need to stay home because of a runny nose. | 8 | 8 | Ich war positiv erstaunt, dass er trotz Krankheit doch so arbeiten geht. | I was pleasantly surprised that he still goes to work despite his illness. | 3 | 8 |
| 34 | Er wirkte trotz Erkältung heiter und ich hatte keine Sorgen zwecks Hygiene, deshalb empfand ich leichtes Mitleid, habe mich aber gefreut, nett bedient worden zu sein. | He seemed cheerful despite his cold and I had no worries about hygiene, so I felt a little pity, but I was glad to have been served nicely. | 6  7 | 6  7 | Dass er wahrscheinlich nicht frei bekommen hat, oder die gröbste Krankheit schon hinter sich hat und wieder arbeiten kann. | That he probably didn't get time off, or the worst of the illness has already passed and he can work again. | 2  3 | 2 |
| 35 | ich empfand es als unverhältnismäßige situation. dienstleistungen eines kranken in Anspruch zu nehmen war mir unangenehm. | I experienced it as an inappropriate situation, making use of the service of an ill person was unpleasant. | 5 | 4 | was wird diese person nach der arbeit machen? ist die ausgangssituation, die wsl zur verstimmung beiträgt u. U. selbst verschuldet? | What will this person do after work? Is the initial situation that is most likely causing the upset possibly self-inflicted? | 8 | 3 |
| 37 | Wollte nicht angesteckt werden | Did not want to get infected. | 2 | 2 | Geh weg nicht das du mich ansteckst | Go away, don't infect me. | 5 | 5 |
| *Continued* | | | | | | | | |
|  |  |  |  |  |  |  |  |  |
|  |  |  |  |  |  |  |  |  |
|  |  |  |  |  |  |  |  |  |
|  |  |  |  |  |  |  |  |  |
|  |  |  |  |  |  |  |  |  |
| Table S4 *Continued* | | | | | | | | |
| No | Original (affective) reactions | Translated (affective) reactions | R1 | R2 | Original thoughts | Translated thoughts | R1 | R2 |
| 38 | Ich war verwundert, dass jemand diesen Job macht, obwohl es ihn sichtlich sehr anstrengt. Im Vergleich zu seinem jüngeren Kollegen sah es eher mühsam aus und wirkte so, als ob bald eine körperliche Grenze erreicht ist. Daher war ich eher beeindruckt, wie schnell alles vonstatten ging und hoffte für den Angestellten, dass sein Chef etwas Rücksicht nimmt. | I was astonished that someone would do this job, even though it obviously takes a lot out of him. Compared to his younger colleague, it looked rather painstaking and seemed as if a physical limit would soon be reached. So I was rather impressed by how quickly everything was done and hoped for the employee that his boss would show some consideration. | 6  9 | 6  7 | Wie kann man unterstützen und sich doch nicht zu sehr in deren Arbeit und Abläufe einmischen. | How can you support them without interfering too much in their work and processes? | 7 | 7 |
| 39 | Bedauerlich | Unfortunate. | 6 | 5 | Wie viele Leute arbeiten, obwohl sie angegriffen sind | How many people are working despite being sick. | 8 | 8 |
| 43 | Mitfühlend | Compassionate. | 6 | 6 | Rückenschmerzen und dann muss er noch so viele Pakete ausliefern | He has to deliver so many packages while having back pain. | 6 | 8 |
| 44 | Die Verkäuferin war nicht ernsthaft krank (im Sinne von: Kurz vorm umfallen) und hat ihren Job trotzdessen gewissenhaft erledigt. | The saleswoman was not seriously ill (in the sense of: about to faint) and she did her job conscientiously regardless. | 8 | 8 | Sympathie, da sie trotz Erkältung arbeitet. | Sympathy, because she works despite her cold. | 8 | 8 |
| 50 | Er wirkte gestresst und kurz angebunden, ich empfand dadurch ein Mitleid dass er vermutlich den Rest seiner Schicht so weiterarbeiten muss. | He seemed stressed and short-tempered, thus I felt pity that he probably have to work the rest of his shift in this way. | 6 | 6 | Dass es nicht fair ist, dass er sich so überarbeiten muss, weil sein Unternehmen bei Gehältern und Mitarbeitern spart um die Gewinne nur in den Chefetagen zu teilen. | That it is not fair that he has to overwork himself like this, because his company is saving on salaries and staff in order to share the profits only in the executive offices. | 1 | 1  2 |
| 52 | Es tat mir ein bisschen leid, vor allem weil es sehr spät war. | I felt a little sorry, especially because it was very late. | 6 | 6 | Ich hätte ihn gern zu mir eingeladen und ihm einen Kaffee angeboten. | I would have liked to invite him to my place and offer him a coffee. | 7 | 7 |
| 54 | Ich habe sie bemitleidet, es tat mir leid und ich habe mich leicht schuldig gefühlt. Gleichzeitig hab ich mich geehrt gefühlt, dass sie das trotzdem unbedingt machen wollte (Termin). | I pitied her, I felt sorry and slightly guilty. At the same time I felt honored that she really wanted to do it anyway (appointment). | 4 | 4  6 | Ich habe mich gefragt, wieso sie trotzdem den ganzen Tag arbeiten kommt und ob es an ihrem Gehalt etc. liegt, dass sie trotzdem kommen muss. | I was wondering why she still comes to work all day and whether it is because of her salary etc. that she has to come anyway. | 2  4 | 2  3 |
| *Continued* | | | | | | | | |
| Table S4 *Continued* | | | | | | | | |
| No | Original (affective) reactions | Translated (affective) reactions | R1 | R2 | Original thoughts | Translated thoughts | R1 | R2 |
| 65 | nicht störend | Not annoying. | 8 | 8 | kenn ich | Familiar to me. | 8 | 8 |
| 67 | Es tat mir leid | I felt sorry. | 6 | 6 | Dass er es nicht leicht hat und sicher sehr viel Stress hat und bestimmt Ärger bekommt, da er Pakete falsch geliefert hat | That he does not have it easy and is certainly under a lot of stress and will certainly get into trouble because he has delivered packages incorrectly. | 6 | 6 |
| 72 | Ich empfand Mitgefühl. | I felt compassioned. | 6 | 6 | Der arme Kerl | That poor guy. | 8 | 8 |
| 75 | Ich hatte Mitleid, wie gerade schon erwähnt, v.a. weil in unserer Gesellschaft sich nicht genug ausgeruht wird und immer gleich weiter gemacht wird, obwohl der Körper einem sagt Geht nicht mehr . Und ich hatte natürlich ein bisschen Angst, dass sie mich ansteckt und sich nicht genug die Hände wäscht oder so ... | I felt pity, as I just mentioned, especially because in our society people are not resting enough and keep going even though the body tells one that it is not working anymore. And of course I was a bit afraid that she might infect me and not wash her hands enough or something ... | 2  6 | 2  6 | / | / | / | / |
| 82 | Irritierend | Confusing [or annoying] | 3 | 3 | Konsumgesellschaft auf dem Rücken der Schwächeren | Consumer society on the back of the weaker. | 1 | 1 |
| 83 | Er tat mir leid | I pitied him. | 6 | 6 | Er braucht Ruhe | He needs rest. | 7 | 7 |
| 84 | Die betreffende Person tat mir leid. | I felt sorry for that person. | 6 | 6 | Tat mir natürlich immer noch leid, fand aber auch ihre Frisur schön. | Of course I still felt sorry for her, but I also liked her hairstyle. | 8 | 8 |
| 89 | Traurig | Sad. | 5 | 5 | Macht der Job einen krank? | Does this job make you sick? | 8 | 4 |
| 90 | Als Zumutung | As an impertinence. | 3 | 3 | Hoffentlich stecke ich mich nicht an | Hopefully I won’t get infected. | 5 | 5 |
| 91 | Fühlte mich schuldig weil ich seine zeit in anspruch nehme. Mitleid dass er sich nicht auskurieren kann | Felt guilty for taking up his time. Pity that he can't cure himself completely. | 4  6 | 4  6 | Er kann es sich scheinbar nicht leisten krank zu sein oder hat angst. Ihm ist arbeit wichtiger als seine Gesundheit. Hat eventuell Angst vor Abmahnung im Job | He apparently cannot afford to be ill or is afraid. Work is more important to him than his health. Maybe he is afraid of receiving a warning letter at work. | 2  3 | 2  3 |
| *Continued* | | | | | | | | |
|  | | | | | | | | |
|  | | | | | | | | |
| Table S4 *Continued* | | | | | | | | |
| No | Original (affective) reactions | Translated (affective) reactions | R1 | R2 | Original thoughts | Translated thoughts | R1 | R2 |
| 94 | Nicht weiter schlimm für die Dienstleistung an sich. | Not bad for the service itself. | 8 | 8 | Jetzt muss sich der arme Kranke auf Arbeit schleppen. | Now the poor sick guy has to drag himself to work. | 8 | 8 |
| 95 | Ich hab gesagt, er solle lieber einen KO-Tag nehmen. Die Chefs sind da sehr kulant. | I told him to take a day of sick leave instead. The bosses are very obliging. | 9 | 9 | Was ihn dazu bringt, auf Arbeit zukommen, trotz Krankheit. | What makes him come to work, despite illness. | 4 | 4 |
| 96 | bedauerlich | Unfortunate. | 6 | 6 | wie könnte man helfen? | How could one help? | 7 | 7 |
| 97 | Abschreckend | Deterrent. | 2 | 2 | Steck mich nicht an | Don't infect me. | 5 | 5 |
| 112 | Unprofessionell | Unprofessional. | 9 | 5 | Der arme Kollege, er muss krank zur Arbeit kommen. | The poor colleague, he has to come to work sick. | 8 | 8 |
| 113 | ich habe direkt danach gefragt, da er lange nicht da war, sondern eine vertretung | I asked for it directly, as he hadn't been there for a long time, but a substitute. | 9 | 9 | / | / | / | / |
| 114 | Es hat mir leidgetan und ich wusste nicht genau, was ich tun oder sagen kann, das angemessen wäre. | I was sorry and I did not know exactly what would have been appropriate to do or say. | 6 | 6 | Warum sie zur Arbeit gekommen ist, ob es aus Angst vor einem Jobverlust ist. | Why she came to work, whether it was out of fear of losing her job. | 2 | 2  4 |
| 119 | Erschreckend | Frightening. | 2 | 5 | Dass ich das System, dass gesundheitlich angeschlagene, alte Menschen dazu zwingt, Pakete in den 5. Stock zu tragen, beschämend finde. | I find the system that forces health-impaired old people to carry parcels to the 5th floor disgraceful. | 1 | 1 |
| 120 | Als bedrückend, unangenehm. | As depressing, unpleasant. | 5 | 4  5 | Paketzuliefer*innen müssen häufig viele Treppen steigen. Sie halten sich in vielen verschiedenen Häusern auf. Häufig kommen Ihnen die Menschen nicht entgegen. Ihre Arbeit wird nicht gewertschätzt. | Employees delivering parcels often have to climb many stairs. They go to many different houses. Often people do not come to meet them. Their work is not appreciated. | 8 | 1 |
| 125 | konnte ich nicht feststellen | I couldn't discern. [or I did not notice.] | 9 | 9 | / | / | / | / |
| *Continued* | | | | | | | | |
|  | | | | | | | | |
|  | | | | | | | | |
|  | | | | | | | | |
| Table S4 *Continued* | | | | | | | | |
| No | Original (affective) reactions | Translated (affective) reactions | R1 | R2 | Original thoughts | Translated thoughts | R1 | R2 |
| 129 | … als nicht weiter störend - jeder Mensch hat mal mit einer Erkältung/ Grippe zu kämpfen. | …not as bothering – everyone has to deal with a cold/flu once in a while. | 8 | 8 | Wenn es der Person erheblich schlechter gegangen wäre, hätte ich vielleicht nachgefragt, warum er sich nicht Zuhause ausruhen würde. Ich dachte mir: Ach, der hat auch eine rote, verschnupfte Nase - ging mir vor ein paar Tagen genauso | If the person had been considerably worse off, I might have asked why he wouldn't rest at home. I thought to myself: "Oh, he has a red, runny nose, too - I felt the same way a few days ago. | 7  8 | 4  8 |
| 133 | Mitelid | Compassion. | 6 | 6 | / | / | / | / |
| 136 | Unangenehm | Unpleasant. | 5 | 5 | Dass Menschen Arbeiten müssen obwohl sie sich nicht wohl fühlen ist unverständlich. Ist durch Ansteckung auch eine Gefahr für Andere | It is incomprehensible that people have to work although they do not feel well. It is also a risk for others to get infected. | 4  5 | 4  5 |
| 139 | Gehustet | Coughed. | 9 | 9 | Sollte zu Hause bleiben, wegen Ansteckungsgefahr, sowie unmittelbarer Arbeit mit Lebensmittel | Should stay at home because of the risk of infection and direct work with food. | 5  7 | 5 |
| 140 | Eklig. Nicht professionell | Disgusting. Unprofessional. | 1 | 1  5 | Muss auch arbeiten, wenn er krank ist. | Needs to work even when he's sick. | 8 | 8 |
| 141 | Menschlich | Humanely. | 9 | 8 | Ansteckungsgefahr durch wechselgeld | Risk of infection due to change [*i.e. money*]. | 5 | 5 |
| 146 | Ich habe nicht darüber nachgedacht, da viele in dieser Zeit angeschlagen waren | I didn't think about it, as many were under the weather at that time. | 8 | 8 | „Es geht wohl wieder was rum“ | "I guess there's a cold going around." | 8 | 8 |
| 151 | Entspannt | Relaxed. | 8 | 7 | Die Arme... | Poor girl. | 8 | 8 |
| 153 | Schlecht | Bad. | 5 | 5 | Mitleid, das eine Krankschreibung anscheinend mit höheren Hürden verbunden ist, Risiko einer Ansteckung | Pity that sick leave seems to be associated with higher hurdles, risk of infection. | 2  5 | 2  5 |
| *Continued* | | | | | | | | |
|  | | | | | | | | |
|  | | | | | | | | |
| Table S4 *Continued* | | | | | | | | |
| No | Original (affective) reactions | Translated (affective) reactions | R1 | R2 | Original thoughts | Translated thoughts | R1 | R2 |
| 154 | Ich hatte Mitleid. | I felt compassion. | 6 | 6 | Dass es ihr hoffentlich bald besser geht. | That she will hopefully get better soon. | 8 | 8 |
| 156 | Nicht schlimm, war ja etwas seelisches was auf dem Weg der Besserung war | Not bad, as it was something mental and the person already recovering. | 8 | 8 | Das ich es mutig finde das der Dienstleister darüber spricht mit mir | That I find it courageous that the service provider talks to me about it. | 8 | 8 |
| 157 | Unangenehm. | Unpleasant. | 5 | 5 | Ich würde vermutlich auch noch arbeiten, ich hatte Mitleid, etwas Ekel (keime), Angst vor Ansteckung | I would probably still work as well, I had pity, some disgust (germs), fear of infection. | 5  8 | 5  8  4 |
| 158 | Furchtbar , aber er meinte, es tue ihm gut und es ist besser als zu Hause zu bleiben | Terrible, but he said it was good for him and better than staying at home. | 5 | 5 | Wie froh man sein kann, wenn man gesund ist.wie lange er das noch machen kann | How happy you can be when you are healthy. How long he can still do this? | 6  8 | 8 |
| 162 | Es tat mir leid, dass die Person trotz ihrer Erkältung arbeiten musste. | I was sorry that the person had to work despite her cold. | 6 | 6 | Dass die Arbeit anstrengend für die Person sein muss. | That the work must be exhausting for the person. | 6 | 6 |
| *Note.* *N* = 54 respondents. No. = serial case number; R1 = Ratings of Rater 1; R2 = Ratings of Rater 2. Coding of the (affective) reactions: 1 – *disgust*, 2 – *fear*, 3 – *anger*, 4 – *guilt*, 5 – *other negative affective reactions*, 6 – *compassion*, 7 – *positive affective reactions*, 8 – *indifference*, 9 – *miscellaneous*. Coding of the thoughts: 1 – *causes of sickness presence in social/economic system*, 2 – *causes of sickness presence in work characteristics*, 3- *individual causes of the employee for sickness presence*, 4 – *unspecific causes for sickness presence*, 5 – *consequences of sickness presence for the customers*, 6 – *consequences of sickness presence for the employee*, 7- *problem oriented coping*, 8 – *miscellaneous*. | | | | | | | | |

| Table S5  *Coding scheme and examples for the respondents’ thoughts about the ill employee (Study 2)* | | | | | |
| --- | --- | --- | --- | --- | --- |
|  | C | Themes | Definition | Exemplary codes | Examples |
| Causes for sickness presence | 1 | Causes in social/ economic system | Reasoning about causes of the behavior (sickness presence) of the employee, especially on causes in society / in the (economic) system | Consumer society on the back of the weaker | *“The system forces people to their knees, sick people should not have to work, the pressure is too high, shitty achievement-oriented society.”* |
|  |  |  |  |  |  |
|  | 2 | Causes in work characteristics | Reasoning about causes of the behavior (sickness presence) of the employee, specifically on causes in the job characteristics or work-related conditions of the employee | Being afraid of a warning at work,  difficult to cancel a shift | *“Understanding towards the service provider, difficult to cancel shifts.”* |
|  |  |  |  |  |  |
|  | 3 | Individual causes of the employee | Reasoning about causes of the behavior (sickness presence) of the employee, especially on causes in the person of the service provider (e.g., attitudes, preferences) or their personal circumstances | Work more important than health,  financial difficulties | *“[…]* *Work is more important to him than his health. […]”* |
|  |  |  |  |  |  |
|  | 4 | Unspecific causes | (Unspecific) reasoning about causes of the behavior (sickness presence) of the employee, reactions that do not fit in the themes 1 to 3 | Why do you have to go to work with a cold? | *“What makes him come to work, despite illness.”* |
|  |  |  |  |  |  |
| Conse-quences of sickness presence | 5 | Consequences for customers | Reasoning about the consequences of the behavior (sickness presence) of the employee for the customer and other customers | Infection, contagion | *“Don’t infect me.”* |
| *Continued* | | | | | |
|  |  |  |  |  |  |
| Table S5 *Continued* | | | | | |
| Main themes | C | Themes | Definition | Exemplary codes | Examples |
|  | 6 | Consequences for employee | Reasoning about the consequences of the behavior (sickness presence) of the employee for the employee her- or himself | Work must be strenuous for the employee, tormented her- or himself,  gets in trouble | *“That he does not have it easy and is certainly under a lot of stress and will certainly get into trouble because he has delivered packages incorrectly.”* |
|  |  |  |  |  |  |
| Problem-oriented coping | 7 | Problem-oriented coping | Reasoning about how employee could be supported or how the situation of the employee could be improved in general | Employee should rest, How could one help? | *“I would have liked to invite him to my place and offer him a coffee.”* |
|  |  |  |  |  |  |
| Miscel-laneous | 8 | Miscel-laneous | Reactions that do not fit in the scheme | I know all about it!,  The poor guy | *“That poor guy.”* |
| *Note.* C = Coding. | | | | | |

Table S6

*Results of the validation of the vignettes used in Study 3*

|  |  | *M* | *SD* | *t* | *p* |
| --- | --- | --- | --- | --- | --- |
| Health Impairments | Scenario 2a | 1.91 | 0.30 |  |  |
|  | Scenario 2b | 4.73 | 0.65 | -12.45 | < .001 |
| *Note*. *N* = 11. The sample included nine women and two men between 21 and 71 years (*M*_age_ = 35.45, *SD* = 15.64) recruited through personal contacts. Participants were asked to rate the probability of the described employee’s medical problems on a 5‑point scale (1 = *very unlikely*, 5 = *very likely*) for each scenario. Both scenarios described a reliable and timely delivery of a parcel (i.e., high service quality). To manipulate employee sickness presence, one of the two scenarios did not include a cue of the employee’s physical illness (Scenario 2a), whereas the other scenario included such a cue (Scenario 2b). | | | | | |

Table S7

*Demographic characteristics for two groups of participants separated by the rated scenario (Study 3)*

|  | Proportion of | |
| --- | --- | --- |
|  | participants rating Scenario 2a | participants rating Scenario 2b |
|  | in % | in % |
| Sex |  |  |
| Women | 75.0 | 71.4 |
| Men | 25.0 | 25.7 |
| Others | / | 2.9 |
| Employment Status |  |  |
| In employment | 37.5 | 40.5 |
| Unemployed | / | 5.7 |
| Retired | / | 2.9 |
| Trainees and students | 56.3 | 45.7 |
| Self-employed | / | 5.7 |
| Industry |  |  |
| Commercial services, trading, distribution, and hospitality | 42.9 | 25.0 |
| Construction and architecture | 18.8 | / |
| Social sciences including linguistics, literature, economics, arts, and culture | 7.1 | 18.8 |
| Logistic, transport, security services | 7.1 | 6.3 |
| Management, accounting, law, and administration | 7.1 | / |
| Medicine and civil services | 14.3 | 31.3 |
| Mining, production, and manufacturing | 7.1 | / |
| Natural sciences, geography and, computer sciences | 7.1 | / |
| Others | 7.1 | / |
| *Note.* Scenario 2a did not include a cue of the employee’s physical illness, whereas Scenario 2b included such a cue. The average age of participants rating Scenario 2a was 29.91 years (*SD* = 11.48, *N*= 36). The average age of participants rating Scenario 2b was 31.63 years (*SD* = 12.04, *N*= 36). | | |

| Table S8  *Direct effects of employee sickness presence on the mediators, the appraisals, and the dependent variables (Study 3)* | | | | | | | | | |
| --- | --- | --- | --- | --- | --- | --- | --- | --- | --- |
|  | Disgust  (*M1*)  *B* (*SE*) | Fear  (*M2)*  *B (SE)* | Anger  (*M3*)  *B* (*SE*) | Guilt  (*M4*)  *B* (*SE*) | Compassion *(M5)*  *B* (*SE*) | Personal  Fairness  *B* (*SE*) | Moral  Fairness  *B (SE)* | Repurchase Intention  *B (SE)* | Recommendation Intention  *B (SE)* |
| Sickness  Presence | 1.18  (0.21)** | 0.39  (0.19)* | 1.21  (0.30)** | 1.37  (0.34)** | 2.95  (0.33)** | -1.39  (0.31)** | -2.16  (0.29)** | -1.22  (0.20)** | -0.86  (0.26)** |
| Constant | 1.05  (0.02)** | 1.23  (0.07)** | 1.05  (0.04)** | 1.29  (0.19)** | 2.74  (0.26)** | 6.26  (0.18)** | 5.12  (0.21)** | 4.53  (0.09)** | 3.94  (0.17)** |
| *R²* | .32** | .06* | .18** | .19** | .55** | .23** | .45** | .35** | .14** |
| *Note*. *N* = 69-72. Sickness Presence: not present (0), present (1); Unstandardized regression coefﬁcients and robust standard errors are reported. **p*< .05; ** *p* < .01. | | | | | | | | | |

| Table S9  *Direct and indirect effects of employee sickness presence on repurchase intention (Study 3)* | | | | | |
| --- | --- | --- | --- | --- | --- |
|  |  | Repurchase Intention  *B* (*SE*) | |  |  |
| Sickness Presence (*X*) |  | -0.87 (0.28)** | |  |  |
| Disgust (*M1*) |  | -0.14 (0.17) | |  |  |
| Fear (*M2*) |  | 0.21 (0.14) | |  |  |
| Anger (*M3*) |  | -0.42 (0.09)** | |  |  |
| Guilt (*M4*) |  | 0.09 (0.09) | |  |  |
| Compassion (*M5*) |  | 0.03 (0.07) | |  |  |
| Constant |  | 4.67 (0.25)** | |  |  |
| *R*² |  | .58** | |  |  |
| Bootstrap indirect effects of *X* on Rep  (through *M1 up to M5*) | | | *B* (*SE*) | LL 95% CI | UL 95% CI |
| Disgust (*M1*) |  | -0.16 (0.19) | | -0.88 | 0.11 |
| Fear (*M2*) |  | 0.08 (0.07) | | -0.03 | 0.24 |
| Anger (*M3*) |  | -0.51 (0.17)* | | -0.87 | -0.20 |
| Guilt (*M4*) |  | 0.13 (0.12) | | -0.12 | 0.37 |
| Compassion (*M5*) |  | 0.09 (0.20) | | -0.29 | 0.49 |
| Total effect of *X* on Rep | | | *B* (*SE*) | LL 95% CI | UL 95% CI |
| Sickness Presence (*X*) | | | -1.24 (0.21)* | -1.65 | -0.83 |
| *Note. N* = 69. Rep = Repurchase Intention; Sickness Presence: not present (0), present (1); LL = lower limit; CI = conﬁdence interval; UL = upper limit; Unstandardized regression coefﬁcients and robust standard errors are reported. Bootstrap sample size = 5,000. * *p* < .05; ** *p* < .01. | | | | | |

| Table S10  *Direct and indirect effects of employee sickness presence on recommendation intention (Study 3)* | | | | |
| --- | --- | --- | --- | --- |
|  |  | Recommendation Intention  *B* (*SE*) |  |  |
| Sickness Presence (*X*) |  | -0.73 (0.40) |  |  |
| Disgust (*M1*) |  | -0.03 (0.17) |  |  |
| Fear (*M2*) |  | -0.10 (0.16) |  |  |
| Anger (*M3*) |  | -0.44 (0.10)** |  |  |
| Guilt (*M4*) |  | 0.05 (0.11) |  |  |
| Compassion (*M5*) |  | 0.15 (0.10) |  |  |
| Constant |  | 1.70 (0.93) |  |  |
| *R*² |  | .36** |  |  |
| Bootstrap indirect effects of *X* on Rec  (through *M1 up to M5*) | | *B* (*SE*) | LL 95% CI | UL 95% CI |
| Disgust (*M1*) |  | -0.04 (0.19) | -0.44 | 0.30 |
| Fear (*M2*) |  | -0.04 (0.06) | -0.16 | 0.07 |
| Anger (*M3*) |  | -0.53 (0.17)* | -0.91 | -0.21 |
| Guilt (*M4*) |  | 0.07 (0.15) | -0.25 | 0.36 |
| Compassion (*M5*) |  | 0.45 (0.28) | -0.14 | 0.97 |
| Total effect of *X* on Rec | | *B* (*SE*) | LL 95% CI | UL 95% CI |
| Sickness Presence (*X*) | | -0.83 (0.26)* | -1.35 | -0.30 |
| *Note. N* = 69. Rec = Recommendation Intention; Sickness Presence: not present (0), present (1); LL = lower limit; CI = conﬁdence interval; UL = upper limit; Unstandardized regression coefﬁcients and robust standard errors are reported. Bootstrap sample size = 5,000. * *p* < .05; ** *p* < .01. | | | | |

| Table S11  *Direct effects of perceived fairness on the dependent variables (Study 3)* | | |
| --- | --- | --- |
|  | Repurchase Intention  *B (SE)* | Recommendation Intention  *B (SE)* |
| Personal Fairness | 0.14 (0.08) | 0.18 (0.10) |
| Moral Fairness | 0.33 (0.08)** | 0.26 (0.09)** |
| Constant | 1.80 (0.39)** | 1.47 (0.48)** |
| *R²* | .41** | .27** |
| *Note*. *N* = 70. Unstandardized regression coefﬁcients are reported. **p*< .05; ** *p* < .01. | | |

Table S12

*Results of the validation of the vignettes used in Study 4*

| Outcomes  Manipulated  Variables | Physical  Distance | Employee  Health  Status | Duration | Affective  Arousal | |
| --- | --- | --- | --- | --- | --- |
| Sickness Presence: 0 – absent;  1 – present | *B* = -0.28, *SE* = 0.27,  *p* = .320 | *B* = -2.77,  *SE* = 0.20,  *p* < .001 | *B* = 0.95,  *SE* = 1.84,  *p*= .610 | *B* = 0.80,  *SE* = 0.74,  *p*= .288 | |
| Physical Distance: 0 – short distance;  1 – long distance | *B* = 1.22,  *SE* = 0.27,  *p* < .001 | *B* = -0.40, *SE* = 0.20,  *p* = .057 | *B*= ‑2.93,  *SE* = 1.84,  *p* = .121 | *B* = -1.08,  *SE* = 0.74,  *p* = .153 | |
| *Note*. *N* = 34. Unstandardized regression coefﬁcients are reported. The sample included 25 women (78.1%) and 6 men (18.8%) with ages between 16 and 97 years (*M*_age_ = 34.29, *SD* = 15.25). Three person did not indicate their gender. Participants were asked to rate the average physical distance between the bank employee and themselves as well as the health status of the bank employee on 5‑point scales (1 = *very short*, 5 = *very long*; 1 = *serious ill-health*, 5 = *in perfect health*) for each scenario. Additionally, they rated the average duration (in minutes) of the described interaction between the bank employee and themselves as well as their level of affective arousal (Bradley & Lang, 1994) in the described situation on 9-point scales (1 = *relaxed, sleepy*, 5 = *stimulated, wide awake*). All four scenarios described a reliable bank service (i.e., high service quality). To manipulate employee sickness presence, two of the four scenarios did not include cues of the bank employee’s physical illness (Scenarios 3a and 4a), while the other scenario included such cues (Scenarios 3b and 4b). To manipulate physical distance between the bank employee and the participant, in two of the four scenarios the interaction took place at the branch of the bank (Scenarios 3a and 3b), while in the other two scenarios the interaction took place via phone call (Scenarios 4a and 4b). | | | | |  |

Table S13

*Demographic characteristics for four groups of participants separated by the rated scenario (Study 4)*

|  | Proportion of | | | |
| --- | --- | --- | --- | --- |
|  | participants rating Scenario 3a | participants rating Scenario 3b | participants rating Scenario 4a | participants rating Scenario 4b |
|  | in % | in % | in % | in % |
| Sex |  |  |  |  |
| Women | 51.0 | 51.3 | 45.5 | 54.3 |
| Men | 49.0 | 48.7 | 54.5 | 45.7 |
| Employment Status |  |  |  |  |
| In employment or self-employed | 56.3 | 65.1 | 67.9 | 72.0 |
| Unemployed | 7.8 | 7.3 | 8.0 | 5.9 |
| Retired | 25.0 | 19.3 | 15.0 | 15.6 |
| Trainees and students | 10.9 | 8.3 | 9.1 | 6.5 |
| Industry |  |  |  |  |
| Agriculture, forestry, animal husbandry, and horticulture | - | 0.8 | 1.4 | 1.3 |
| Commercial services, trading, distribution, and hospitality | 20.2 | 25.4 | 21.3 | 20.4 |
| Construction and architecture | 6.5 | 2.3 | 6.4 | 5.3 |
| Social sciences including linguistics, literature, economics, arts, and culture | 3.2 | 1.5 | 1.4 | 3.3 |
| Logistic, transport, security services | 4.8 | 5.4 | 8.5 | 8.6 |
| Management, accounting, law, and administration | 4.0 | 6.9 | 7.1 | 7.9 |
| Medicine and civil services | 19.4 | 24.6 | 21.3 | 21.7 |
| Mining, production, and manufacturing | 8.9 | 6.2 | 5.7 | 4.6 |
| Natural sciences, geography and, computer sciences | 2.4 | 5.4 | 2.8 | 5.9 |
| Others | 30.6 | 21.5 | 24.1 | 21.1 |
| *Note.* The average age of participants rating Scenario 3a was 47.72 years (*SD* = 15.88). The average age of participants rating Scenario 3b was 46.74 years (*SD* = 15.44), while average age of participants rating Scenario 4a was 44.43 years (*SD* = 14.52). The average age of participants rating Scenario 4b was 45.02 years (*SD* = 13.99). | | | | |

| Table S14  *Direct and indirect effects of employee sickness presence on repurchase intention (Study 4)* | | | | | |
| --- | --- | --- | --- | --- | --- |
|  |  | Repurchase Intention  *B* (*SE*) | |  |  |
| Sickness Presence (*X*) |  | -0.56 (0.17)** | |  |  |
| Disgust (*M1*) |  | -0.12 (0.06)** | |  |  |
| Fear (*M2*) |  | 0.13 (0.07) | |  |  |
| Anger (*M3*) |  | -0.35 (0.07)** | |  |  |
| Guilt (*M4*) |  | 0.16 (0.07)* | |  |  |
| Compassion (*M5*) |  | 0.18 (0.05)** | |  |  |
| Constant |  | 4.11 (0.14)** | |  |  |
| *R*² |  | .41** | |  |  |
| Bootstrap indirect effects of *X* on Rep  (through *M1 up to M5*) | | | *B* (*SE*) | LL 95% CI | UL 95% CI |
| Disgust (*M1*) |  | -0.27 (0.14)* | | -0.54 | -0.01 |
| Fear (*M2*) |  | 0.13 (0.07) | | -0.01 | 0.28 |
| Anger (*M3*) |  | -0.55 (0.11)* | | -0.77 | -0.34 |
| Guilt (*M4*) |  | 0.07 (0.03)* | | 0.01 | 0.13 |
| Compassion (*M5*) |  | 0.41 (0.10)* | | 0.22 | 0.61 |
| Total effect of *X* on Rep | | | *B* (*SE*) | LL 95% CI | UL 95% CI |
| Sickness Presence (*X*) | | | -0.77 (0.12)* | -1.01 | -0.53 |
| *Note. N* = 763. Rep = Repurchase Intention; Sickness Presence: not present (0), present (1); LL = lower limit; CI = conﬁdence interval; UL = upper limit; Unstandardized regression coefﬁcients and robust standard errors are reported. Bootstrap sample size = 1,000. * *p* < .05; ** *p* < .01. | | | | | |

| Table S15  *Direct and indirect effects of employee sickness presence on recommendation intention (Study 4)* | | | | |
| --- | --- | --- | --- | --- |
|  |  | Recommendation Intention  *B* (*SE*) |  |  |
| Sickness Presence (*X*) |  | -0.57 (0.17)** |  |  |
| Disgust (*M1*) |  | -0.15 (0.07)* |  |  |
| Fear (*M2*) |  | 0.14 (0.06)* |  |  |
| Anger (*M3*) |  | -0.31 (0.07)** |  |  |
| Guilt (*M4*) |  | 0.10 (0.07) |  |  |
| Compassion (*M5*) |  | 0.19 (0.04)** |  |  |
| Constant |  | 4.34 (0.13)** |  |  |
| *R*² |  | .17** |  |  |
| Bootstrap indirect effects of *X* on Rec  (through *M1 up to M5*) | | *B* (*SE*) | LL 95% CI | UL 95% CI |
| Disgust (*M1*) |  | -0.33 (0.14)* | -0.45 | -0.04 |
| Fear (*M2*) |  | 0.13 (0.06)* | 0.02 | 0.25 |
| Anger (*M3*) |  | -0.49 (0.10)* | -0.68 | -0.27 |
| Guilt (*M4*) |  | 0.04 (0.03) | -0.02 | 0.10 |
| Compassion (*M5*) |  | 0.44 (0.09)* | 0.25 | 0.63 |
| Total effect of *X* on Rec | | *B* (*SE*) | LL 95% CI | UL 95% CI |
| Sickness Presence (*X*) | | -0.77 (0.12)* | -1.00 | -0.54 |
| *Note. N* = 763. Rec = Recommendation Intention; Sickness Presence: not present (0), present (1); LL = lower limit; CI = conﬁdence interval; UL = upper limit; Unstandardized regression coefﬁcients and robust standard errors are reported. Bootstrap sample size = 1,000. * *p* < .05; ** *p* < .01. | | | | |

| Table S16  *Direct effects and interaction effects of employee sickness presence and physical distance on the appraisals (Study 4)* | | | | | | |
| --- | --- | --- | --- | --- | --- | --- |
|  | Goal Congruence  *B* (*SE*) | Agency Employee  *B (SE)* | Agency Management  *B* (*SE*) | Agency Customer  *B* (*SE*) | Situational Control  *B* (*SE*) | Certainty  *B (SE)* |
| Sickness Presence (*X*) | -1.08 (0.15)** | 0.12 (0.14) | 0.32 (0.16)* | -1.43 (0.16)** | 0.03 (0.16) | -0.44 (0.17)** |
| Physical Distance (*Mod*) | 0.05 (0.15) | -0.44 (0.14)** | -0.09 (0.16) | 0.24 (0.15) | 0.29 (0.16) | -0.17 (0.17) |
| *X*Mod* | 0.73 (0.22)** | -0.18 (0.21) | 0.09 (0.23) | 0.00 (0.23) | 0.30 (0.23) | 0.64 (0.24)** |
| Constant | 5.27 (0.10)** | 4.96 (0.10)** | 4.25 (0.11)** | 4.47 (0.10)** | 2.85 (0.11)** | 4.13 (0.12)** |
| *R²* | .08** | .04* | .02** | .18** | .02** | .01* |
| *Note*. *N* = 754-761. Sickness Presence: not present (0), present (1); Physical Distance: short (0), long (1); Unstandardized regression coefﬁcients and robust standard errors are reported. **p*< .05; ** *p* < .01. | | | | | | |

| Table S17  *Direct effects of the appraisals on the dependent variables (Study 4)* | | |
| --- | --- | --- |
|  | Repurchase Intention  *B (SE)* | Recommendation Intention  *B (SE)* |
| Goal Congruence | 0.63 (0.03)** | 0.52 (0.03)** |
| Agency Employee | -0.05 (0.04) | -0.02 (0.04) |
| Agency Management | 0.04 (0.03) | -0.03 (0.03) |
| Agency Customer | 0.10 (0.03)** | 0.12 (0.03)** |
| Situational Control | 0.08 (0.03)** | 0.07 (0.03)* |
| Certainty | 0.01 (0.03) | 0.08 (0.03)* |
| Constant | 0.04 (0.27) | 0.64 (0.28)* |
| *R²* | .40** | .33** |
| *Note*. *N* = 754. Unstandardized regression coefﬁcients are reported. **p*< .05; ** *p* < .01. | | |
